# Positional ^13^C enrichment analysis of aspartate determines PEPC activity *in vivo*

**DOI:** 10.1111/nph.70412

**Published:** 2025-07-24

**Authors:** Luisa Wittemeier, Yogeswari Rajarathinam, Alexander Erban, Martin Hagemann, Joachim Kopka

**Affiliations:** ^1^ Max‐Planck Institute of Molecular Plant Physiology Am Mühlenberg 1 14476 Potsdam Germany; ^2^ Environmental and Biochemical Sciences The James Hutton Institute Invergowrie Dundee DD2 5DA UK; ^3^ Department of Plant Physiology University of Rostock Albert‐Einstein‐Straße 3 18059 Rostock Germany

**Keywords:** aspartate, CO_2_ assimilation, C‐positional E^13^C analysis, dynamic ^13^C labeling, GC‐MS, PEPC, RUBISCO, *Synechocystis* sp. PCC 6803

## Abstract

- Photoautotrophic organisms fix inorganic carbon (Ci) by RIBULOSE‐1,5‐BISPHOSPHATE CARBOXYLASE/OXYGENASE (RUBISCO) and PHOSPHOENOLPYRUVATE CARBOXYLASE (PEPC). Monitoring Ci assimilation rates *in vivo* is a major challenge in analyzing photoautotrophic metabolism and engineering improved photosynthesis, as conventional methods do not distinguish between these two fluxes.
- We explored widely applied gas chromatography mass spectrometry (GC‐MS) metabolite profiling for C‐positional fractional ^13^C enrichment (E^13^C) analyses of aspartate to differentiate within one molecule between PEPC, RUBISCO, and CBB cycle activities by ^13^C pulse‐labeling. We validated this method using two GC‐MS instruments and two prevailing chemical derivatization methods.
- We selectively determined E^13^C at each carbon position of aspartate with accuracy < 1% and precision < 2.5%. In combination with dynamic ^13^CO_2_ labeling of *Synechocystis* cultures, we determined PEPC activity *in vivo* alongside assessments of RUBISCO and CBB cycle activities. We demonstrate that RUBISCO is inactive in the dark, whereas PEPC remains active but at a lower rate than during the day.
- Accurate quantifications of aspartate concentrations and positional E^13^Cs provide molar Ci assimilation rates of photoautotrophic *Synechocystis* cultures. This technology can be combined with C‐positional analyses of other metabolites, for example 3‐phosphoglycerate, and may be adapted to characterize natural and biosynthetically engineered Ci‐assimilation.

## Introduction

Metabolic flux analyses reveal changes in metabolism by isotopic labeling. ^13^C labeling and analysis by gas chromatography mass spectrometry (GC‐MS) are techniques that examine the production and consumption rates of metabolites. Labeling can either be analyzed at isotopic steady‐state to verify contributions of different carbon sources (Kruger *et al*., 2012), that is, the alternative uses of metabolic pathways and reactions, or together with dynamic flux studies (Geffen *et al*., 2023), in which concentration and labeling information of metabolites are monitored at consecutive timepoints after a stable isotope pulse. In photoautotrophic metabolism, CO_2_ is the only carbon substrate that ultimately leads to uniform labeling at metabolic steady‐state (Jazmin & Young, 2013). By contrast, dynamic flux studies monitor the rates of label incorporation (Fernie & Morgan, 2013) where the initial labeling can be non‐uniform depending on enzyme reaction mechanisms. However, measurements of ^13^C enrichment into a complete molecule do not allow direct insight into the source of carbon assimilation into this molecule if more than one reaction contributes to its formation.

Positional ^13^C labeling information is crucial to understand metabolic pathway regulation and to estimate enzyme activities *in vivo*. Positional information after ^13^C labeling can be directly obtained by nuclear magnetic resonance spectroscopy (NMR) (Zamboni *et al*., 2009). This versatile quantitative technology is limited by low sensitivity and requires large amounts of biological material. GC‐MS is equally widely established for metabolomic analyses, offers higher sensitivity, and provides information at isotopologue level. MS determines how many carbons of a molecule are labeled, but does not allow direct conclusions on positional labeling and the presence of defined isotopomers, that is, isomers with identical isotopic composition but isotopic atoms at different positions of the structure (Chatham *et al*., 2003; Ratcliffe & Shachar‐Hill, 2006). *In silico* fragmentation analysis of positional labeled tricarboxylic acid (TCA) cycle metabolites analyzed by gas chromatography‐electron impact ionization‐mass spectrometry (GC‐EI‐MS) and GC‐EI‐tandem mass spectrometry (GC‐EI‐MS/MS) suggested several fragment ions (fragments) that are useful for positional fractional ^13^C enrichment (E^13^C) analysis (Okahashi *et al*., 2019). Several previous studies showed that calculation of positional E^13^C is possible using GC‐EI‐MS measurements of amino acids, organic acids, or glucose (Lima *et al*., 2021; Dellero *et al*., 2023; Daubermann *et al*., 2024; Rajarathinam *et al*., 2025).

Photoautotrophic organisms such as cyanobacteria, algae, and plants use oxygenic photosynthesis to produce reducing power (NADPH) and energy (ATP) by light reactions. The products of the light reactions drive inorganic carbon (Ci) fixation and production of organic biomass. In cyanobacteria, Ci, in the forms of CO_2_ or HCO_3_^−^, enters metabolism via two main carboxylation reactions that are catalyzed by RIBULOSE‐1,5‐BISPHOSPHATE CARBOXYLASE/OXYGENASE (RUBISCO) and PHOSPHOENOLPYRUVATE CARBOXYLASE (PEPC) (Durall & Lindblad, 2015). RUBISCO, the key enzyme of the Calvin‐Benson‐Bassham (CBB) cycle, assimilates CO_2_ using ribulose‐1,5‐bisphosphate (RuBP) and generates two molecules of 3‐phosphoglycerate (3PGA). PEPC fixes HCO_3_^−^ using phosphoenolpyruvate (PEP) and forms oxaloacetate (OAA) and inorganic phosphate. OAA is rapidly converted to malate, aspartate, or citrate by MALATE DEHYDROGENASE (MDH), ASPARTATE AMINOTRANSFERASE (AAT) or CITRATE SYNTHASE (CS), respectively (Mills *et al*., 2020). The carbon backbone of OAA and of the downstream metabolites, aspartate or malate, comprises four carbon atoms. RUBISCO assimilates Ci (CO_2_) into the 1‐C position of 3‐phosphoglycerate (3PGA). The Calvin‐Benson‐Bassham (CBB) cycle redistributes fixed carbon atoms to the 2,3‐C_2_ of 3PGA. PEPC uses phosphoenolpyruvate derived from 3PGA and assimilates Ci (HCO_3_^−^) into the 4‐C of oxaloacetate (OAA) (Kai *et al*., 2003). 1,2,3‐C_3_ of OAA and of its transaminase product aspartate originate from 1,2,3‐C_3_ of PEP and maintain the carbon configuration of 3PGA from Ci assimilation via RUBISCO and the CBB cycle.

In land plants, PEPC is crucial for C_4_ and crassulacean acid metabolism (CAM) photosynthesis (Keeley & Rundel, 2003). Malate or aspartate serve as transport metabolites in variants of C_4_ metabolism (Schlüter & Weber, 2020). CAM photosynthesis uses malate to build a temporal carbon store in vacuoles during the night that delivers CO_2_ to RUBISCO by the action of MALIC ENZYME during the day (Heyduk, 2022). Through these mechanisms, Ci is concentrated in the vicinity of RUBISCO and the wasteful RUBISCO oxygenase reaction is suppressed. In C_3_ plants, PEPC has an alternative key function by catalyzing the anaplerotic synthesis of OAA to replenish TCA cycle intermediates and balancing carbon and nitrogen metabolism (Melzer & O'Leary, 1987; Shi *et al*., 2015). In addition to RUBISCO, PEPC can contribute to Ci assimilation in cyanobacteria (Tabita, 1994). For example, PEPC has a substantial contribution to CO_2_ assimilation yielding 25% of total Ci assimilation in *Synechocystis* sp. PCC 6803 (*Synechocystis*) under mixotrophic conditions, as was suggested by metabolic flux studies based on ^13^C‐glucose labeling (Yang *et al*., 2002) or 12% under photoautotrophic conditions, as was predicted by genome‐scale isotopic non‐stationary metabolic flux analysis (Gopalakrishnan *et al*., 2018). PEPC is an essential enzyme in *Synechococcus* PCC 7942 and *Synechocystis* (Luinenburg & Coleman, 1990; Angermayr *et al*., 2014). PEPC orthologs of more evolved cyanobacterial clades, such as Oscillatoriales and Nostocales, have characteristic features that are comparable to C_4_ isoforms, whereas more basal groups of cyanobacteria, such as Chroococcales and Pleurocapsales, have neither dedicated C_3_ nor C_4_ isoforms (Shylajanaciyar *et al*., 2015).

PEPC activity can be determined *in vitro* by spectrophotometric assays that are coupled to MDH and LACTATE DEHYDROGENASE and are based on measurements of NADH reduction (Meyer *et al*., 1988). *In vitro* activity assays do not account for cellular cofactors, regulators, substrate availability, effects of subcellular structures, or metabolic channeling. Hence, those tests do not reflect cytosolic *in vivo* conditions completely. To address this analytical gap, we assessed PEPC activity *in vivo* by mass spectrometry. Direct analysis of OAA is demanding due to its very low cellular concentration and its rapid conversion into downstream metabolites, for example, aspartate, malate, and citrate (Hasunuma *et al*., 2018; Ito *et al*., 2021). In land plants, positional isotopomer analysis of malate by either NMR or GC‐MS was used as a proxy for PEPC activity *in vivo* (Abadie & Tcherkez, 2019; Lima *et al*., 2021). However, flux balance analysis suggested that the main flux in *Synechocystis* leads from OAA via AAT to aspartate (Knoop *et al*., 2013). Non‐stationary metabolic flux analyses detected flux in photoautotrophic conditions through MDH, AAT, and CS, whereby MDH and AAT seem to catabolize OAA to equivalent amounts (Young *et al*., 2011; Gopalakrishnan *et al*., 2018). The modelled ranges of fluxes through many enzyme reactions were, however, still very wide (Young *et al*., 2011; Gopalakrishnan *et al*., 2018). *Synechocystis* CS has low catalytic activity and seems to be an inefficient enzyme causing low flux through the oxidative branch of the TCA cycle (Ito *et al*., 2019).

Previously, GC and liquid chromatography (LC) coupled to MS/MS allowed determination of the complete isotopomer distribution of aspartate (Choi *et al*., 2012; Cai *et al*., 2023). Here, we applied routine direct in‐source fragmentation of GC‐MS systems which is technically and analytically less complex. We predicted aspartic acid fragmentation *in silico* and confirmed predictions through analysis of positional labeled aspartic acid reference substances in combination with exact mass determinations. These analyses were enabled by a GC‐atmospheric pressure chemical ionization‐MS (GC‐APCI‐MS) instrument operated at high mass resolution and compared to a GC‐EI‐MS operating at nominal mass resolution. Limitations of detection and accuracy of ^13^C enrichment measurements were evaluated based on mixtures of positional labeled and natural aspartic acid. In combination with dynamic ^13^CO_2_ labelling of *Synechocystis*, we determined position specific carbon assimilation into each carbon atom of aspartate and assessed 4‐C as a proxy of PEPC activity *in vivo* alongside contributions of RUBISCO and CBB cycle activities to 1‐C, 2‐C, and 3‐C during day and night conditions. Our method proves the expected absence of RUBISCO activity in the dark and changing PEPC activity upon day‐to‐night shift.

## Materials and Methods

### Aspartic acid standard mixtures

Natural aspartic acid, fully labeled [U‐^13^C]‐aspartic acid and positionally labeled [1‐^13^C]‐, [2‐^13^C]‐, [3‐^13^C]‐, [4‐^13^C]‐aspartic acid were purchased from Sigma Aldrich/Merck KGaA (Darmstadt, Germany), according to analytical certificates of the manufacturer with natural, 99.0, 99.6, 99.7, 99.6, and 99.3% ^13^C enrichment of the complete molecules and at the single carbon positions, respectively. Mixtures with different ratios of natural and all positionally labeled aspartic acids were prepared in aqueous solution at molar ratios of 5 : 95, 10 : 90, 50 : 50, 90 : 10, or 95 : 5 and dried by vacuum centrifugation (Supporting Information Table S1). The dried mixtures were used to assess the accuracy of E^13^C determination at 25 ng per GC‐MS injection as the mean deviation of measured E^13^C from expected E^13^C and the precision of E^13^C measurements as the SD of E^13^C deviation. To determine the lower detection threshold of E^13^C measurements, positionally labeled aspartic acid standards were mixed in a molar ratio of 1 : 1 : 1 : 1. This mixture was diluted by natural aspartic acid to 100, 50, 20, 10, and 4% of total aspartic acid (Table S1). Different amounts of the isotope‐diluted mixtures were analyzed by GC‐MS at 1.25, 2.5, 12.5, 25, 125, and 250 ng per injection. All samples were spiked with 25 ng per injection of ^13^C_6_‐sorbitol (Sigma‐Aldrich/Merck KGaA, 99 atom% ^13^C, 99% chemical purity) as a constant internal standard.

### Cultivation and sampling of *Synechocystis* sp. PCC 6803

Glucose‐tolerant *Synechocystis* wild‐type (WT) precultures were grown for 3 d at 30°C in modified BG11 growth medium (Rippka *et al*., 1979). To avoid an additional carbon source, citric acid and ferric ammonium citrate were replaced by a 0.021 mM FeCl_3_ iron source. *Synechocystis* cells were cultivated in a Multicultivator MC 1000‐OD photobioreactor (Photon Systems Instruments, Drásov, Czech Republic) under continuous illumination set to 100 μmol photons m^−2^ s^−1^ and with high inorganic carbon supply, that is, constant bubbling (2 bubbles × s^−1^) with 5% CO_2_ in air. After pre‐cultivation, culture media were renewed by centrifugation and resuspension of cells. Illumination was changed to 12 h light : 12 h dark cycles, maintaining 100 μmol photons m^−2^ s^−1^ in the light phases. After three day : night cycles, medium was exchanged and OD_750_ adjusted to *c*. 0.8 in the third light phase 4 h before the onset of darkness by fast vacuum filtration with continuous illumination. Sampling of cells was at and after transition to the fourth night by < 15 s filtration onto glass fiber filters (25 mm, pore size 1.6 μm; Cytiva, Sigma Aldrich/Merck KGaA) and immediate shock freezing in liquid N_2_. First sampling at t_0_ was harvested in the light with 5% ambient CO_2_ bubbling. Before dynamic ^13^CO_2_ labeling at the onset of darkness, the culture medium was exchanged by fast filtration to remove dissolved non‐labeled Ci from the cultures. Cells were resuspended in the dark by immediate bubbling with 5% ^13^CO_2_ in synthetic air. Labeled samples at t_1_–t_6_ were collected 5, 10, 15, 30, 60, and 90 min after the beginning of ^13^CO_2_ bubbling. OD_750_ × mL^−1^ was adjusted to *c*. 1.0 and recorded of all individual cultures at t_0_, t_1_, and t_6_. For continuous light cultivation and dynamic ^13^CO_2_ labeling in the light, the procedure was identical, omitting only the change to day‐night cycles.

### Metabolite extraction

Polar metabolites were extracted from deep frozen cells on filters using methanol (Sigma‐Aldrich, gradient grade for liquid chromatography, ≥ 99.9%) chloroform (Sigma‐Aldrich/Merck KGaA, contains ethanol as stabilizer, ACS reagent grade, ≥ 99.8%) and double distilled water (ddH_2_O). One milliliter of extraction mix, that is methanol : chloroform : ddH_2_O (2.5 : 1 : 1; v/v/v) with 6 μg mL^−1 13^C_6_‐sorbitol as internal standard, was added and incubated for 15 min at 70°C under permanent agitation. Phase separation was induced by adding 400 μl of ddH_2_O. The upper polar phase (*c*. 800 μl) was separated by centrifugation and dried overnight by vacuum centrifugation (Scheurer *et al*., 2021).

### Chemical derivatization for GC‐MS analysis

Chemical derivatization of dried metabolite samples for GC‐MS analysis was exactly as described previously, omitting 4‐(dimethylamino)pyridine (Erban *et al*., 2020). Samples were subjected to methoxyamination followed by either trimethylsilylation (TMS) using *N,O*‐bis(trimethylsilyl)trifluoroacetamide (BSTFA, Macherey‐Nagel, Düren, Germany) or using the same protocol, replacing trimethylsilylation with *tert*.‐butyldimethylsilylation (TBDMS) with *N*‐methyl‐*N*‐(*tert*.‐butyldimethylsilyl)trifluoroacetamide (MTBSTFA, Macherey‐Nagel) reagent and an incubation for 60 min at 70°C followed by 15 min at 30°C under permanent agitation.

### GC‐MS analysis

Derivatized samples were analyzed by an Agilent 6890 N24 gas chromatograph (Agilent Technologies, Waldbronn, Germany) hyphenated to either electron impact ionization‐time of flight‐mass spectrometry (EI‐TOF‐MS) using a LECO Pegasus III time of flight mass spectrometer (LECO Instrumente GmbH, Mönchengladbach, Germany) or to atmospheric pressure chemical ionization‐time of flight‐mass spectrometry (APCI‐TOF‐MS) with a micrOTOF‐Q II hybrid quadrupole time‐of‐flight mass spectrometer (Bruker Daltonics, Bremen, Germany) equipped with an APCI ion source and GC interface (Bruker Daltonics) (Kopka *et al*., 2017). All measurements were conducted in splitless mode using a 5% phenyl – 95% dimethylpolysiloxane fused silica capillary column with 30 m length, 0.25 mm inner diameter, 0.25 μm film thickness, and an integrated 10 m precolumn (Agilent Technologies (CP9013)). Measurements of chemical standard mixtures were conducted with additional paired injections onto the GC‐APCI‐MS system using a split ratio of 1 : 5 (v/v) with TMS‐derivatized samples and a split ratio of 1 : 100 (v/v) with TBDMS samples. Retention index standardization was based on *n*‐alkanes as described earlier (Erban *et al*., 2020).

### Quantification of aspartic acid

GC‐EI‐MS chromatograms were recorded at nominal mass resolution, baseline corrected, and processed as described previously (Erban *et al*., 2020). Chemical reference compounds and their analytes were annotated by manual supervision using TagFinder (Luedemann *et al*., 2011) and the NIST MS Search 2.0 software (http://chemdata.nist.gov/). Observed experimental mass spectra and retention time indices (RI) were matched to the mass spectral and RI reference collection of the Golm Metabolome Database (GMD) (Kopka *et al*., 2005). A######‐### identifiers are GMD entry codes. Quantification of isotopologues and isotopologue distributions (MIDs) was based on peak apex abundances.

Exact masses of GC‐APCI‐MS files were internally calibrated based on PFTBA (Kopka *et al*., 2017). Files were transcribed into mzXML format using Bruker DataAnalysis and AutomationEngine software (v.4.2). Analytes of GC‐APCI‐MS files were identified manually based on exact monoisotopic masses, comparison to the paired GC‐EI‐MS analyses, and parallel measurements of metabolite reference compounds. The isotopologue abundances of molecular ions and fragments with respective ^13^C labeled MIDs were extracted from each GC‐APCI‐MS file in a defined chromatographic time range adjusted to each analyte and in a mass range of ±0.005 mass units using the R packages xcms (v.3.22.0) (Tautenhahn *et al*., 2008), MSnbase (v.2.26.0) (Gatto *et al*., 2021), and msdata (v.0.40.0) (Neumann & Gatto, 2017) in RStudio (2023.6.1.524, http://www.posit.co/, R v.4.3.1). Quantification of isotopologue abundances was based on the area under the peak apex ±10 scans.

For metabolite quantification, the sum of all isotopologue abundances was used. Metabolite concentration was normalized to the internal standard ^13^C_6_‐Sorbitol, OD_750_ of the cell culture, and sample volume. Molar metabolite concentrations were acquired through parallel analysis of calibration series of non‐labeled reference compounds. Mass spectra and chromatogram plots were generated by Bruker DataAnalysis.

### Determination of ^13^C enrichment

MIDs and resulting ^13^C enrichment calculations of mass features were corrected for natural isotope abundances (NIA) according to their specific molecular formula using RStudio and the IsoCorrectoR package (v.1.18.0) (Heinrich *et al*., 2018). Correction of tracer impurity was done manually by adjusting IsoCorrectoR results to the specified ^13^C purity of each reference substance. E^13^C and molar concentrations of aspartic acid were used to calculate molar ^13^C concentration at each carbon position of the aspartic acid backbone.

### Calculation of positional ^13^C enrichment

Position‐specific ^13^C enrichment of aspartic acid for position 1, 2, 3, and 4 was calculated according to Eqns (Eqn 1), (Eqn 2), (Eqn 3), (Eqn 4), respectively:

(Eqn 1) $$E^{13}C\left(1‐C\right)=4\times E^{13}C\left(1,2,3,4‐C\right)-3\times E^{13}C\left(2,3,4‐C\right)$$

(Eqn 2) $$E^{13}C\left(2‐C\right)=3\times E^{13}C\left(2,3,4‐C\right)-2\times E^{13}C\left(3,4‐C\right)$$

(Eqn 3) $$E^{13}C\left(3‐C\right)=\begin{array}{l} 2\times E^{13}C\left(2,3‐C\right)+2\times E^{13}C\left(3,4‐C\right)-3\\ \times \;E^{13}C\left(2,3,4‐C\right) \end{array}$$

(Eqn 4) $$E^{13}C\left(4‐C\right)=3\times E^{13}C\left(2,3,4‐C\right)-2\times E^{13}C\left(2,3‐C\right)$$

Mass features of aspartic acid analytes from GC‐MS analyses that contain required carbon combinations, 1,2,3,4˗C, 2,3,4˗C, 2,3˗C, and 3,4˗C and their use for position‐specific ^13^C enrichment calculations are reported in the results section.

### Estimation of molar carbon assimilation by sigmoidal curve fitting

Assimilation rates in 1‐C and 4‐C of aspartate were determined by sigmoidal curve fitting using R Studio and package sicegar (v.0.2.4, threshold_AIC = −10 (default setting)) (Caglar *et al*., 2018). Absolute positional E^13^C (pmol × OD_750_^−1^ × mL^−1^) at 0, 5, 10, 15, 30, 60, and 90 min served as input data. The threshold intensity ratio was set to 0.75 and the maximum allowed intensity at t_0_ to 0. The maximum slope in the sigmoidal plot equals the maximum assimilation rate (pmol × OD_750_^−1^ × mL^−1^ × min^−1^) and the time point (min) when maximum assimilation is reached is called ‘half max’.

## Results and Discussion

### Exploration of GC‐MS technology towards ^13^C‐positional enrichment analysis of aspartate

To assess *in vivo* activity of PEPC, we first aimed to measure steady state amounts of OAA and its products malate or aspartate. Malate and aspartate were reliably quantified as reported before (Huege *et al*., 2011; Orf *et al*., 2015), whereas we did not obtain measurements of OAA from extracts of *Synechocystis*. To check the detection limit of our methods, we subjected different amounts of OAA to GC‐EI‐MS and GC‐APCI‐MS analyses. The GC‐EI‐MS system did not detect OAA below 30 ng injected using the main analyte 1MEOX 2TMS MP (A147010‐101) (Fig. S1). The limit for GC‐APCI‐MS was 1 ng OAA. This amount corresponds to a detectable concentration of ≥ 0.1 nmol OAA × OD_750_^−1^ × mL^−1^ in *Synechocystis* cells when 10 ml of culture are sampled at OD_750_ = 1 (or ≥ 3.0 nmol × OD_750_^−1^ × mL^−1^ when analyzed by GC‐EI‐MS). Cellular concentrations of OAA were lower likely due to its immediate conversion to product metabolites. Malate concentrations in *Synechocystis* extracts were close to the lower detection limit of our GC‐MS systems. Because flux studies predicted a main flux from OAA to aspartate with minor fluxes to malate and citrate in *Synechocystis* (Young *et al*., 2011; Knoop *et al*., 2013), we decided to use positional labeling information from aspartate as a proxy of Ci assimilation modes in *Synechocystis*.

To determine positional E^13^C information of the aspartate carbon backbone, we analyzed the fragmentation patterns of aspartic acid molecules derivatized by two different derivatization reagents, BSTFA and MTBSTFA, that are commonly used for GC‐MS analyses of primary metabolites and amino acids. These reagents introduce TMS (SiC_3_H_9_) or TBDMS (SiC_5_H_15_) modifications at carboxy‐, hydroxy‐, thiol‐, amino‐, and amide‐groups, and thereby make the resulting derivatized molecules amenable to GC analyses. We identified the aspartic acid derivatives within GC‐MS chromatograms by matches to retention indices and mass spectra of authenticated reference compounds from the Golm Metabolome Database, http://gmd.mpimp‐golm.mpg.de (Kopka *et al*., 2005) and by new reference measurements of ^13^C‐labeled and non‐labeled aspartic acid standard compounds (Fig. S2). All hydroxy‐ and carboxy‐groups of aspartic acid were silylated. Silylation of the amino‐group was partial and generated two detectable aspartic acid derivatives, named in the following aspartic acid 2TMS and 3TMS or 2TBDMS and 3TBDMS, respectively. The analytes with 3 silyl groups were more abundant in standard measurements or biological extracts. Aspartic acid 3TMS, after BSTFA derivatization, was chosen for the quantification of compound abundance. For ^13^C enrichment determination, all available analytes were tested because we expected that the isotopologue distributions are not affected by analyte abundances.

**Fig. 1:**
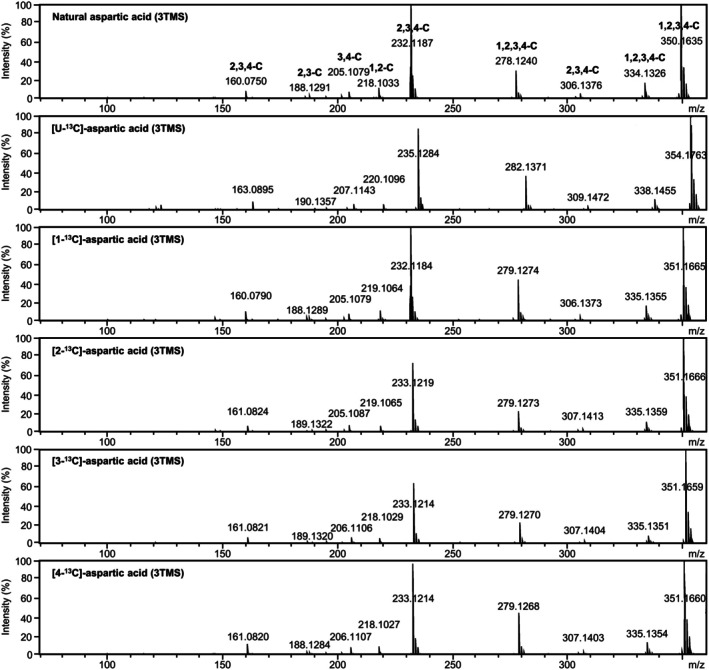
Atmospheric pressure chemical ionization (APCI)‐induced fragmentation of aspartic acid 3TMS. Natural, fully, and position‐specific ^13^C‐labeled aspartic acids were trimethylsilylated (TMS) and analyzed by gas chromatography (GC) coupled to APCI‐mass spectrometry (MS). Representative mass spectra of the aspartic acid 3TMS derivatives are displayed. Mass shifts of fragments from fully labeled [U‐^13^C]‐aspartic acid indicate the numbers of included carbon atoms originating from aspartic acid. Mass shifts of fragments from positionally labeled aspartic acids provide C‐positional information of the C‐atoms present within each fragment. Fragment interpretations are indicated within the mass spectrum of natural aspartic acid (top). All fragment abundances are intensities (%) normalized to the base peak abundance of each mass spectrum (*m/z*, mass to charge ratio).

First, all measured mass features of the silylated aspartic acid derivatives were screened for the presence of its 4 carbon atoms. Mass spectra of fully labeled [U‐^13^C]‐aspartic acid in comparison to mass spectra of natural aspartic acid ascertained the number of carbon atoms originating from aspartic acid within each fragment by mass shift analyses (Fig. 1). Labeled [1‐^13^C], [2‐^13^C], [3‐^13^C], and [4‐^13^C]‐aspartic acids determined the presence of the specific positional carbon atoms (Fig. 1). These analyses demonstrated fragments with carbon combinations 1,2,3,4‐C, 2,3,4‐C, 1,2‐C, 2,3‐C, and 3,4‐C of aspartic acid (Fig. 1). In most cases, GC‐APCI‐MS and GC‐EI‐MS detected fragments with the same nominal mass to charge ratios (*m/z*) (Fig. S3). Only a few differences of fragmentation patterns originated from the ionization modes of the two GC‐MS technologies. GC‐APCI‐MS can be considered a soft ionization method with a higher yield of molecular ions, for example, [M + H]^+^, compared to GC‐EI‐MS ionization that causes rapid dissociation reactions of the initially generated radical molecular ion [M]^+^ (Figs 1, S3–S5) (Li *et al*., 2015). Neutral losses caused by GC‐APCI‐MS (Strehmel *et al*., 2014) induced in‐source fragmentation reactions that matched largely to GC‐EI‐MS, for example aspartic acid 3TMS (Figs 1, S3), aspartic acid 2TMS (Fig. S4), and aspartic acid 3TBDMS (Fig. S5).

The high mass accuracy of the GC‐APCI‐MS system determined molecular masses of fragments with high precision. Therefore, we used the GC‐APCI‐MS analyses to suggest and validate molecular formula predictions of all observed fragments. Knowledge of the molecular formulas is necessary for natural isotope abundance (NIA) correction and determination of E^13^C. Combining C‐positional information of the labelled aspartic acids and the matches of exact mass with predictions from *in silico* fragmentation analyses (Fig. S6), we suggested molecular formulas of most of the fragments detected by GC‐APCI‐MS and GC‐EI‐MS. To predict GC‐APCI‐MS fragmentations, we assumed initial protonation [M + H]^+^, followed by sequential neutral eliminations, intramolecular rearrangements, or intermolecular transfer reactions. These reactions take place after GC within the ionization device, that is, ‘in‐source’, and before MS analysis of all ions that retain a positive charge. GC‐EI‐MS technology generates molecular radical ions [M]^+^ that rapidly dissociate in‐source into neutral radicals and positively charged fragment ions (McLafferty & Turecek, 1993). After dissociation, neutral eliminations and intramolecular rearrangements can follow. Molecular formulas of fragments were determined manually, considering all mechanisms of in‐source ion fragmentation (McLafferty & Turecek, 1993). Recently published software tools, such as the MS Interpreter (https://chemdata.nist.gov/dokuwiki/doku.php?id=chemdata:interpreter) or QCEIMS (Grimme, 2013; Wang *et al*., 2020) can support manual interpretation. All identified and interpreted fragments from this study are listed in Table S2 together with their measured properties.

### Positional purity of fragments

Mass features from GC‐MS only provide information with positional specificity if the fragments and their MIDs are monitored without isobaric interference. Such interferences can be caused either by other fragments of the respective aspartic acid derivative or by co‐eluting compounds from complex sample matrices. The later interferences may arise with every change of experimental conditions and always need to be considered carefully. To test for the presence of interferences, reference compounds with known E^13^C and fragments with known molecular formulas can be analyzed for deviations of expected from measured E^13^C. Previous studies used either precisely labeled standards (Choi *et al*., 2012; Lima *et al*., 2021) or biological samples with controlled stable isotope label (Millard *et al*., 2014; Dellero *et al*., 2023). We used four commercially available, positionally ^13^C‐labeled aspartic acid standards in combinations with non‐labeled aspartic acid (Figs 1, S3–S5) and performed paired analyses by GC‐EI‐MS and GC‐APCI‐MS to characterize the positional purity of fragments and define the lower detection limit of E^13^C determinations.

**Fig. 2:**
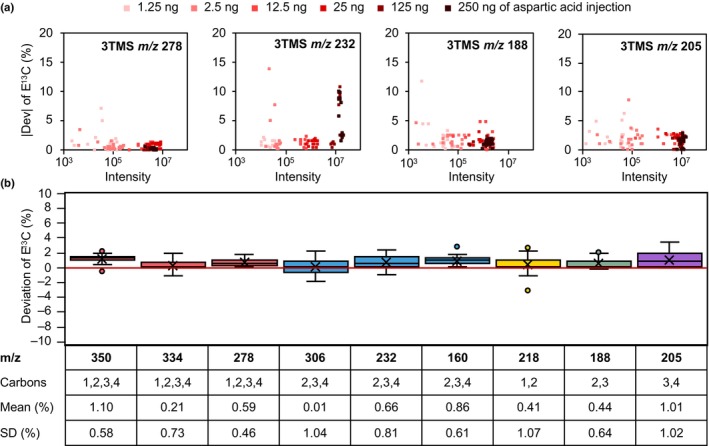
Accuracy and precision of fractional ^13^C enrichment (E^13^C) determination of aspartic acid 3TMS fragments and adducts analyzed by gas chromatography (GC)‐atmospheric pressure chemical ionization (APCI)‐mass spectrometry (MS). Equal mixtures of four positional ^13^C‐labeled aspartic acid standards in different isotopic dilutions with natural aspartic acid and at different concentrations (Supporting Information Table S1) were measured by GC‐APCI‐MS. Deviations of E^13^C, that is measured E^13^C subtracted from expected E^13^C (|Dev|), of the specified fragment were analyzed. (a) Absolute values of E^13^C deviations depend on the specific abundances, that is the sum of all isotopologue abundances, of the selected fragments and on the amounts of injected aspartic acid. Most fragments provide accurate enrichment information with injections ≥ 12.5 ng aspartic acid. (b) Box‐plot representation of E^13^C deviations with means and SD of selected fragments. Fragments are defined by nominal mass to charge ratio (*m*/*z*) and included carbon atoms of aspartic acid (left to right: red, 1,2,3,4‐C; blue, 2,3,4‐C; yellow, 1,2‐C; green, 2,3‐C; purple, 3,4‐C). Thirty‐six different mixtures were analyzed by four technical replicates each at 25 ng aspartic acid injected. The mean and SD provide accuracy and precision information, respectively. All fragments were analyzed by GC‐APCI‐MS measurements in split‐less mode, except fragments *m*/*z* 350 and *m*/*z* 232 that were acquired by split mode injections at split ratio 1 : 5 to avoid interference by detector saturation (standard box plot elements: x, average; boxes, 75^th^ percentile, median, and 25^th^ percentile; whiskers, 100^th^ percentile and 0^th^ percentile; outliers, circles).

E^13^C accuracy can depend on the amount of injected substance and may be compromised by chemical or electronic noise at the lower limit or by detector saturation at the upper limit of detection. To find these limits of E^13^C determination, all positional aspartic acid standards were combined equally and then isotopically diluted by natural aspartic acid in different proportions (Table S1). Injections of 1.25 ng up to 250 ng of each mixture were analyzed to cover the abundance range of our analyses in agreement with the expected biological variation (Orf *et al*., 2015; Scheurer *et al*., 2021). Most fragments showed small absolute deviations from expected E^13^C when 12.5 ng or more were injected (Fig. 2a). Fragment *m/z* 232, which was close to base peak abundance, showed an increase of absolute E^13^C deviation for injections > 125 ng and fragment abundance > 10^7^ (arbitrary units). This saturation phenomenon was fragment specific and applied to fragment *m*/*z* 350, too. Split injection measurements by GC‐MS with adjusted split ratios can be performed when the abundance of aspartic acid should be saturated. All subsequent analyses of fragments *m*/*z* 232 and *m*/*z* 350 were with adjusted split ratios (Fig. 2b).

To determine positional accuracy of E^13^C information of fragments, the positional ^13^C‐labeled aspartic acid standards were mixed with natural aspartic acid in different ratios (Table S1). Sample injection corresponded to optimized 25 ng of aspartic acid, that is the sum of labeled and non‐labeled aspartic acids. E^13^C was determined using predicted molecular formulas (Table S2). The deviation of the determined E^13^C from the expected E^13^C was calculated (Fig. 2b). We used the mean deviation and the SD of the mean deviation as quality parameters for accuracy and precision, respectively. Low values indicate high accuracy and precision. For aspartic acid 3TMS analyzed by GC‐APCI‐MS, all fragments had mean deviations as well as SDs of the mean deviation < 1.1%. Accuracy and precision are in the range of deviations found before when analyzing positional labeled aspartic acid standards by GC‐MS/MS (Choi *et al*., 2012). The comparably high deviation of fragment *m*/*z* 350, which is the [M + H]^+^ adduct ion, originated from the presence of the molecular ion [M]^+^ with overlapping MIDs. If no other fragments comprising the whole carbon backbone should be available, E^13^C of *m/z* 350 can be corrected for the superimposed MID of *m*/*z* 349 ([M]^+^) using a previously reported correction function, CorMID (Langenhan *et al*., 2022).

In the analyzed range of injected sample amounts (Fig. 2a), detector saturation was only observed for GC‐APCI‐MS, not for GC‐EI‐MS (Fig. S7). The deviation of E^13^C was mostly higher when using GC‐EI‐MS, likely due to the lower mass resolution of the instrument and the resulting inferior separation of fragments and isotopologues from overlapping interferences. Acceptable fragments of aspartic acid 3TMS from these paired GC‐EI‐MS analyses only covered carbon combinations 1,2,3,4‐C, 2,3,4‐C, and 1,2‐C, that are insufficient to calculate E^13^Cs of all C‐positions of aspartic acid. GC‐EI‐MS required higher concentrated samples. Low abundant fragments gained accuracy when injections were ≥ 125 ng. Aspartic acid 2TMS, as an alternative but lower abundant option, fragments less compared to aspartic acid 3TMS when analyzed by GC‐EI‐MS. When monitored by GC‐APCI‐MS, fragments of aspartic acid 2TMS again covered carbon combinations 1,2,3,4‐C, 2,3,4‐C, and 1,2‐C (Fig. S8). However, aspartic acid 3TMS measured by GC‐APCI‐MS provided fragments that represented 1,2,3,4˗C, 2,3,4˗C, 2,3˗C, and 3,4˗C. These data allow the monitoring of all C positions from aspartic acid and the combination of E^13^C information from aspartic acid 3TMS and 2TMS if detectable within the same samples. Aspartic acid 3TBDMS fragmentation by GC‐APCI‐MS or by GC‐EI‐MS alone did not cover all fragment combinations necessary to calculate complete positional E^13^Cs (Fig. S9) of aspartic acid. Aspartic acid 2TBDMS was only detectable by GC‐APCI‐MS.

All fragments detected by this study through GC‐EI‐MS and/or GC‐APCI‐MS analyses are listed and characterized (Table S2). We confirmed all inferred molecular formula and excluded interferences of E^13^C measurements by complete or partial overlaps of measured MIDs. Overlaps can be caused by other fragment ions of equal *m*/*z* or of small *m*/*z* differences that are not resolved by the MS instruments at hand. These isobaric interferences may originate from alternative fragments of the aspartic acid derivatives, as well as from co‐eluting laboratory contaminations that are caused by impurities of the materials, solvents, and reagents required for GC‐MS analysis. The extraction background and the sample matrix of complex biological extracts will add additional sources of isobaric interferences. Both need to be considered and interferences excluded by non‐sample controls and non‐labeled samples that are generated under the conditions of planned stable isotope pulse labeling experiments. The minimum validation requirement should be that NIA correction of the used mass features from extracts of non‐labeled samples approximate E^13^C = 0. Fragments should be chosen based on isobaric purity, that is, the absence of overlaying interferences, and based on abundance that should be within the optimized limits of E^13^C quantification. The analysis ranges of complex biological extracts can be characterized using non‐labeled samples that are tested at increasing injection amounts as exemplified (Fig. 2a). Saturation at the upper detection limits can be compensated both by reanalysis in split injection mode or, if necessary, by dilution of the chemically derivatized samples with silylation reagent.

### Calculation of C‐positional E^13^C

Positional E^13^C can be calculated using linear equations combining information of different fragments in a similar way as used before to determine positional E^13^C of glucose, glutamate, and malate (Lima *et al*., 2021). We developed equations that subtract E^13^C among fragment ions, for example, subtracting E^13^C of fragment A with a smaller number of C‐atoms from E^13^C of fragment B with a larger number of C‐atoms, weighted by the number of C‐atoms present in these fragments. The resulting E^13^C relates to the C‐atom(s) that are in this example present in fragment B but not in A. The Eqns (Eqn 1), (Eqn 2), (Eqn 3), (Eqn 4) allow calculation of all positional E^13^C based on available fragments of aspartic acid 3TMS that represent 1,2,3,4˗C, 2,3,4˗C, 2,3˗C, and 3,4˗C. For E^13^C of 1‐C, 2‐C, and 4‐C, E^13^Cs of two fragments are necessary. E^13^C of the 3‐C position required three fragments, because the available in‐source fragmentation did not provide a simpler option. The equations are weighted by the number of carbon atoms that are present in the monitored fragments, because the E^13^C of a molecule with multiple C‐atoms is equal to the average E^13^C across all single C‐positions. Consequently, to determine the E^13^C of a single C‐position from two fragments that differ in the number of carbon atoms, the calculations need to be weighted by the fragments' carbon numbers. We developed this strategy because fragments that represent single C‐atoms are rare and typically low abundant within in‐source fragmentation spectra. Direct means of positional measurements were not available in the case of aspartic acid TMS analytes (Figs 2, S7, S8; Table S2).

**Fig. 3:**
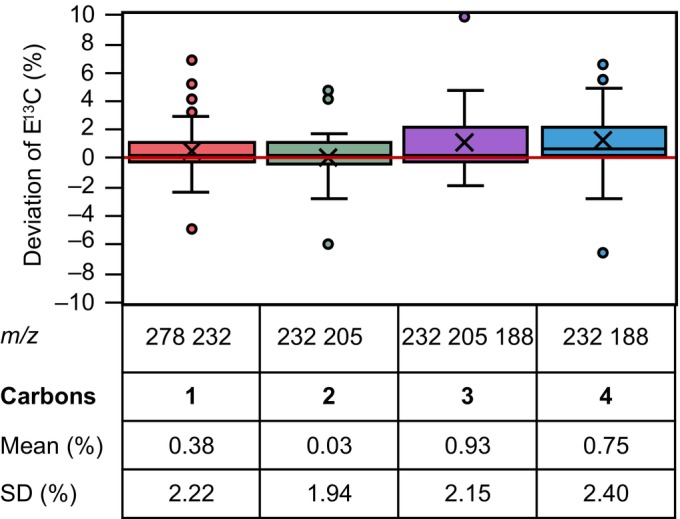
Positional fractional ^13^C enrichment (E^13^C) accuracy and precision of aspartic acid 3TMS determined by gas chromatography (GC)‐atmospheric pressure chemical ionization (APCI)‐mass spectrometry (MS). Positional E^13^C was calculated by Eqns (Eqn 1), (Eqn 2), (Eqn 3), (Eqn 4) using E^13^C of the indicated fragments. Distribution analyses of E^13^C deviations, that is, calculated E^13^C subtracted from expected, are displayed across 36 different mixtures with four technical replicates. The amount of total aspartic acid injected was 25 ng. E^13^C can be determined at all positions with accuracy (mean) < 1% and precision (SD) < 2.5%. E^13^C of fragment *m/z* 232 was determined by split measurements at split ratio 1 : 5. E^13^Cs of all other fragments were determined in split‐less mode. Left to right, 1‐C of aspartic acid (red), 2‐C of aspartic acid (green), 3‐C of aspartic acid (purple), and 4‐C of aspartic acid (blue) (*m*/*z*, mass to charge ratio; standard box plot elements: x, average; boxes, 75^th^ percentile, median, and 25^th^ percentile; whiskers, 100^th^ percentile and 0^th^ percentile; outliers, circles).

Accuracy and precision of positional E^13^C calculations were tested using the standard measurements introduced in the previous section. Accuracy of E^13^C was high for all C‐positions when analyzing aspartic acid 3TMS by GC‐APCI‐MS (Fig. 3), and in the range of the direct E^13^C measurements of single fragments (Fig. 2b). As was expected, SD of E^13^C deviation increased due to error propagation by using initial E^13^C of 2 or 3 fragments. This technical error of positional E^13^C determination was below the biological variation demonstrated later in our studies (see subsequently) and was deemed acceptable for interpretations of biological data.

Multiple alternatives exist to determine positional E^13^C by GC‐APCI‐MS or GC‐EI‐MS, and TMS or TBDMS derivatization (Figs S10–S12). Alternative calculations of positional E^13^C can be used as internal sanity checks provided these measurements are not subject to derivative‐ or fragment‐specific interferences. Such interferences were apparent in some of our combinatorial calculations that in part had decreased accuracy and precision (Figs S10–S12). For this reason, we advise against non‐supervised integration of all available data and suggest instead choosing the best performing combinations after careful validation of the respective instrumentation and interferences of the biological object under investigation.

In detail, aspartic acid 2TMS contributed 3 alternative fragments *m/z* 262, 245, and 160, representing 1,2,3,4‐C or 2,3,4‐C, with E^13^C accuracy and precision similar to our choices from aspartic acid 3TMS (Figs 2b, 3). Combinatorial calculations of E^13^C with fragments of both, aspartic acid 2TMS and aspartic acid 3TMS (Fig. S10), did not improve positional E^13^C determinations.

GC‐EI‐MS determined E^13^C of 3 C‐positions, excluding 3‐C of aspartic acid, but not all at equal accuracy (Fig. S11). Accuracy of two selected combinatorial E^13^C calculations of 1‐C and 2‐C was acceptable, but less precise (Fig. S11A,C). 4‐C E^13^C was consistently underestimated (Fig. S11B) likely due to interference of fragment *m*/*z* 188.

TBDMS derivatization combined with GC‐APCI‐MS measurements determined the positional E^13^C of all positions from aspartic acid with acceptable accuracy but only by combining information from the two analytes, aspartic acid 3TBDMS and aspartic acid 2TBDMS (Fig. S12). Precision was, however, inferior compared to TMS derivatization. The two TBDMS derivatized fragments that directly represented 2‐C of aspartic acid had approximately equal precision compared to the calculations based on TMS derivatization (Figs 3, S12B).

In conclusion, the accuracy and precision of positional E^13^C determinations by in‐source mass fragmentations depended on the choice and number of available fragments and derivatives. Considering potential error propagation by our combinatorial calculations, we argued that fragments with the least mean E^13^C deviation and smallest SD will provide the best positional E^13^C information (Fig. 3). For biological applications, we suggest to first assess all available primary E^13^C information from the multiple options that we provide, to check for interferences of the biological material and the inherent technical variability using given available instrumentation, and only then choose the best available combination for C‐positional calculations. As judged by the *Synechocystis* samples of this study, we found the aspartic acid 3TMS analyte (Fig. 3) superior to the less abundant fragments of aspartic acid 2TMS that were frequently at its lower detection limit. We decided on TMS derivatization because TBDMS derivatives had equal or lower precision.

### Determination of PEPC activity *in vivo*

**Fig. 4:**
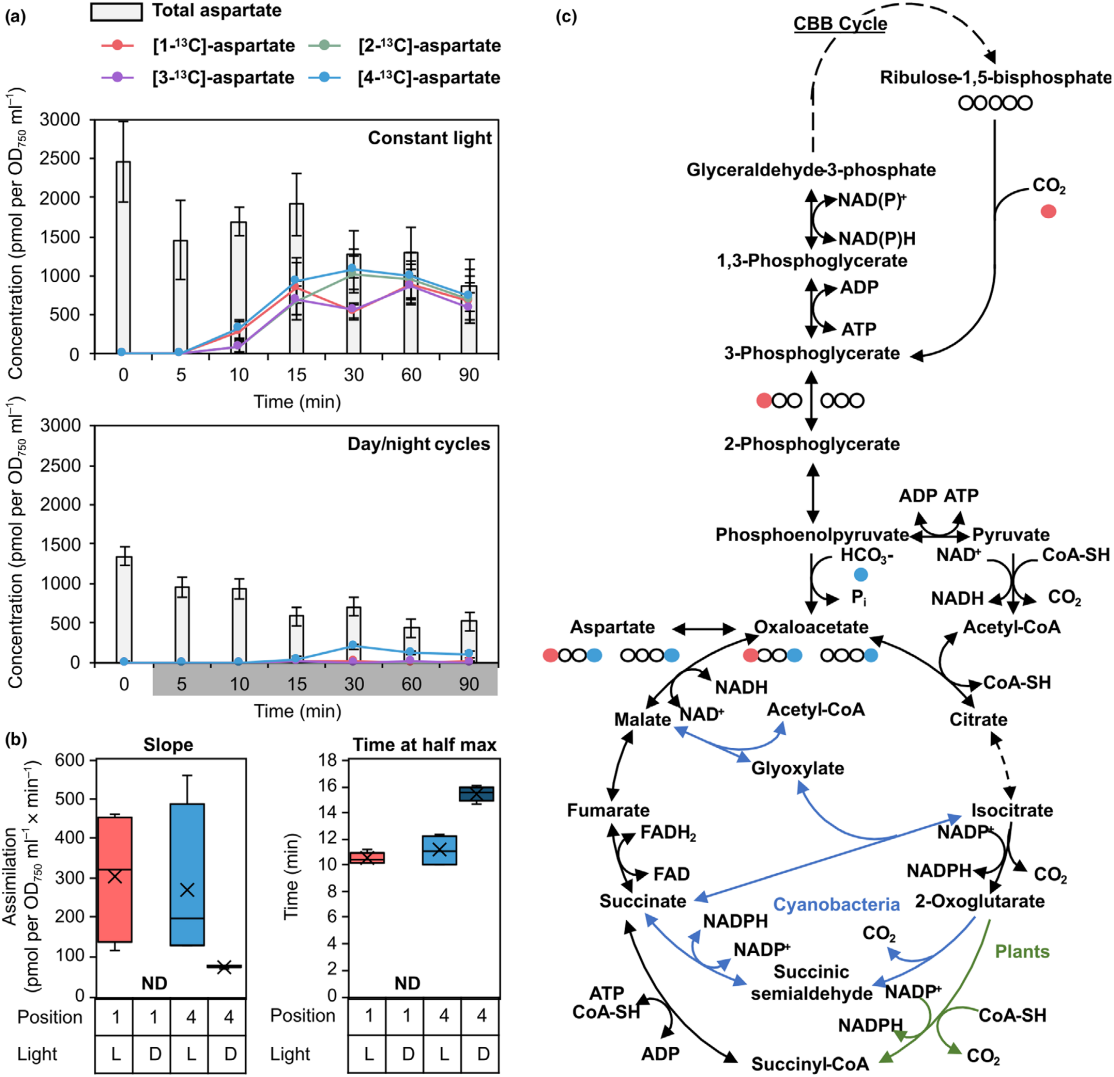
Determination of position‐specific ^13^C labelling of aspartate from *Synechocystis* sp. PCC 6803 during the day compared to the night. *Synechocystis* cells were cultivated photoautotrophically with 5% CO_2_‐enriched air. Cells were either cultivated in constant light or in a 12 h : 12 h day : night cycle. For the day/night cycle experiments, the ^13^CO_2_ labelling pulse was applied directly after transition to darkness. Samples were taken at 5–90 min after the labelling pulse. Samples were analyzed by gas chromatography (GC)‐mass spectrometry (MS) as was described in this study. (a) Aspartate concentrations (pmol × OD_750_^−1^ × mL^−1^) were determined (grey bars). Position‐specific fractional ^13^C enrichment (E^13^C) was multiplied with aspartate concentrations (lines and markers). Data are the means of four biological replicates ± SD. During the day, PEPC and RUBISCO are active. All carbon positions of aspartate assimilate ^13^C. In the night, only PEPC is active; ^13^C assimilation is exclusive to position 4‐C. (b) To estimate molar carbon‐assimilation rates catalyzed by PEPC and RUBISCO, we fitted the positional ^13^C assimilation curves to sigmoidal functions (Supporting Information Fig. S13). Maximum carbon assimilation (slope at half maximal labelling) and the timepoint of maximum assimilation (time at half maximal labelling) are displayed for positions 1‐C and 4‐C in the light (L) and in darkness (D). (c) Metabolic map of selected reactions from central carbon and TCA metabolism of *Synechocystis*. Rows of circles represent the carbon backbone of 3PGA, OAA and aspartate. Filled circles represent the fate of labeled carbon atoms assimilated by RUBISCO (red) or PEPC (blue). Dashed arrows represent multiple reaction steps. Black arrows are reactions common to photoautotrophic organisms, blue arrows are reactions that can occur in cyanobacteria, green arrows are plant specific reactions. Note, cyanobacteria do not have an oxoglutarate dehydrogenase (Smith *et al*., 1967). This reaction is replaced by two enzymes, oxoglutarate decarboxylase and succinic semialdehyde dehydrogenase, which generate succinate via succinic semialdehyde typically at low flux rates (Zhang & Bryant, 2011). The glyoxylate shunt exists in some cyanobacteria (Zhang & Bryant, 2015) but likely not in *Synechocystis* (Knoop *et al*., 2013). In plants it is compartmentalized (ND, not detected; standard box plot elements: x, average; boxes, 75^th^ percentile, median, and 25^th^ percentile; whiskers, 100^th^ percentile and 0^th^ percentile; outliers, circles; 3PGA, 3‐phosphoglycerate; ADP, adenosine diphosphate; ATP, adenosine triphosphate; CBB cycle, Calvin‐Benson‐Bassham cycle; CoA‐SH, coenzyme A; FAD and FADH_2_, oxidized and reduced forms of flavin adenine dinucleotide; HCO_3_^−^, bicarbonate; NAD(P)^+^ and NAD(P)H, oxidized and reduced forms of nicotinamide adenine dinucleotide (phosphate); OAA, oxaloacetate; PEPC, PHOSPHOENOLPYRUVATE CARBOXYLASE; Pi, inorganic phosphate; RUBISCO, RIBULOSE‐1,5‐BISPHOSPHATE CARBOXYLASE/OXYGENASE; TCA, tricarboxylic acid).

The established C‐positional method was combined with dynamic ^13^CO_2_ labeling of *Synechocystis* to estimate PEPC activity and to distinguish this activity from RUBISCO and CBB cycle activities. *Synechocystis* WT was cultivated with high CO_2_ supply (5%) and either in constant light or after entrainment to day/night cycles where ^13^CO_2_ labeling was started in the dark at the beginning of the night. Positional E^13^Cs were determined as described before by analyzing aspartic acid 3TMS within complex extracts of primary metabolites. In constant light, all positions of aspartate were ^13^C labeled with the first detectable E^13^C at 10 min after the ^13^CO_2_ pulse (Fig. 4a). As was expected, our data demonstrate that both PEPC and RUBISCO assimilate CO_2_ and incorporate these carbon atoms into aspartate during the day. E^13^C at 1‐C of aspartate originates directly from CO_2_ assimilation by RUBISCO into 1‐C of 3PGA (Fig. 4c). Label at 2‐C and 3‐C of aspartate and 3PGA results from regeneration of RuBP from 3PGA through the CBB cycle.

At the beginning of the night, first ^13^C label incorporation into aspartate was detected after 15 min. ^13^C label was exclusively delimited to the 4‐C position of aspartate. We concluded that only PEPC was active in the dark and RUBISCO inactive. Our experiments were in full agreement with expectations of PEPC and RUBISCO activity during day and night phases and confirmed that our method indeed distinguished both enzyme activities that contribute to aspartate biosynthesis. We did not detect transfer of ^13^C between carbon positions 1‐C and 4‐C of aspartate during the night. Such a transfer is potentially conceivable (Fig. 4c) via OAA conversion into malate and a malate to fumarate equilibrium catalyzed by fumarase (Abadie & Tcherkez, 2019; Katayama *et al*., 2019). The symmetric fumarate molecule allows position‐swapping between 1‐C and 4‐C and may redistribute ^13^C label, if the reversed reaction sequence from fumarate to OAA should be active. *In vitro* studies, however, demonstrated that the *K*_m_ (Michaelis–Menten constant) for the generation of malate from fumarate is higher than the *K*_m_ of the reverse reaction (Katayama *et al*., 2019). In addition, a fivefold higher ^13^C atom number, that is μmol (^13^C) × g (dry cell weight)^−1^, was present in malate than in fumarate *in vivo* (Hasunuma *et al*., 2018). These studies together indicated that fumarase acts preferentially unidirectionally. Furthermore, flux balance analyses showed that the TCA cycle is operated in a reductive direction with no cyclic flux during the day (Fig. 4c). This operation‐mode of the TCA cycle prevents shuffling of ^13^C label between C‐positions (Steuer *et al*., 2012; Knoop *et al*., 2013). Even though a low cyclic flux was predicted during the night (Steuer *et al*., 2012), no redistribution of label between 4‐C and 1‐C was detected in our studies. An alternative cyclic flux through the glyoxylate shunt (Fig. 4c) that is present in plants and some cyanobacteria (Zhang & Bryant, 2011) is thought to be absent from *Synechocystis*, based on biochemical and modeling studies (Knoop *et al*., 2013). Therefore, we concluded that E^13^C at 4‐C of aspartate is a specific proxy of PEPC‐mediated carbon assimilation.

*In vivo* PEPC activity depends on enzyme amount per cell, the inherent specific enzyme activity, the regulatory state, posttranslational modifications, and availability of the substrates, PEP and HCO_3_^−^. To characterize the CO_2_ assimilation, we calculated molar concentrations, that is pmol of total, ^13^C labeled, or non‐labeled carbon atoms × OD_750_^−1^ × mL^−1^ by quantifications of aspartate amounts using paralleled GC‐EI‐MS measurements and quantitative calibration of the analyses by dilution series of pure aspartic acid reference. Quantifications of E^13^C by GC‐APCI‐MS and of molar amounts by GC‐EI‐MS were paired by sample and allowed quantifications of ^13^C labeled molar amounts at each C‐position of aspartate across our dynamic ^13^CO_2_ labeling experiments (Fig. 4a). The resulting time series of molar quantities were subjected to sigmoidal curve fitting to estimate the maximal C‐assimilation rate into carbon positions of aspartate. For this purpose, we determined the maximum slope of sigmoidal functions fitted over time (Fig. S13). The assimilation rates through RUBISCO into 1‐C and through PEPC into 4‐C of aspartate during the day were similar, indicating that C‐assimilation into aspartate was coordinated and balanced between both enzymes (Fig. 4b). We observed that the variation between replicate time series was caused by light‐dependent variation of aspartate concentration measurements rather than by SD of E^13^C (Fig. 4a). Measurements of assimilation rates through PEPC into 4‐C of aspartate in darkness had low variability (Fig. 4b).

To interpret the positional carbon assimilation rates into aspartate via RUBISCO, that is 1‐C of 3PGA, and via the CBB cycle, that is 2‐C and 3‐C of 3PGA through regeneration of RuBP, dilution of 3PGA by anaplerotic reactions from non‐labeled carbohydrate sources needs to be considered (Makowka *et al*., 2020). Likewise, OAA can be generated from extant non‐labeled malate. Therefore, we consider assimilation rates into 1‐C and 4‐C of aspartate as proxies of enzyme activities that are not directly comparable between positions due to different numbers of metabolic reaction steps between the respective enzyme activities and the aspartic acid pool. However, comparison of assimilation rates into the same position can be compared between different conditions, strains, or mutants. We demonstrated that the assimilation rate of PEPC is lower during the night than during the day, and, as was expected, RUBISCO assimilation was not detectable in darkness. The time point of maximal C positional assimilation is a second characteristic of the monitored enzyme activities. During the night, more time was needed to reach maximal assimilation rates (Fig. 4b). This observation was also in agreement with previous knowledge. The prolonged time until maximum labeling rates is likely due to reduced CO_2_ uptake into *Synechocystis* cells during the night as Ci uptake systems are known to be inactivated in darkness (Price *et al*., 2008).

## Conclusions

Positional E^13^C analyses of aspartate by GC‐MS instruments without MS^2^ capabilities provide positional E^13^C and accurate molar quantifications simultaneously. Positional E^13^C analysis of aspartate can be combined and extended by analyses of other metabolites that are detectable within the same chromatograms, for example 3PGA (Rajarathinam *et al*., 2025), using GC‐MS based profiling technology. The metabolite profiling technology has been designed more than two decades ago with the intention of analyzing concentration changes of as many primary metabolites as feasible (Fiehn *et al*., 2000; Lisec *et al*., 2006). This method has since been continuously and widely applied to phenotype primary metabolism. With the current and previous (Rajarathinam *et al*., 2025) developments, we extend the use towards C‐positional flux analyses and enable re‐analyses of previous labeling studies that did not yet consider C‐positional aspects. Moreover, GC‐MS technology requires less sample material compared to ^13^C positional NMR analysis, and instruments are less costly. Resolving the contribution of different reactions to the carbon backbone of aspartate helps to clarify metabolic routes and allows specification and higher precision of future flux analyses. In photoautotrophic organisms, such as cyanobacteria, Ci assimilation by RUBISCO, the recycling process of RuBP via the CBB cycle, and Ci assimilation by PEPC can be distinguished by the method established in this study. The analysis pipeline extends conventional methods, such as CO_2_ gas exchange measurements (Busch *et al*., 2024) or uptake measurements of heavy ^13^C (e.g. López‐Sandoval *et al*., 2018) or radioactive ^14^C (e.g. Steemann Nielsen, 1952; Timm *et al*., 2015) into total biomass, that do not distinguish the proportions of CO_2_ uptake via these reactions. The technology established in this study can be extended to the various other metabolites that are simultaneously detectable by GC‐MS profiling and is only constrained by the compound specific in‐source fragmentation reactions (e.g. Rajarathinam *et al*., 2025).

The technological features of *in vivo* Ci assimilation assessments through PEPC will be especially powerful when all C‐assimilating reactions are active, for example in photoautotrophic growth conditions, and when combined with direct *in vivo* measurements of RUBISCO assimilation by positional E^13^C analysis of 1‐C from 3PGA within the same samples (Rajarathinam *et al*., 2025). Our method is ready to analyze central carbon metabolism of different regulatory mutants or genetic cyanobacteria backgrounds under changing environmental conditions (Mills *et al*., 2020; Lucius & Hagemann, 2024). Beyond this field, adaptations to analyses of natural and engineered phototrophic algae and plants will open up the direct characterizations of synthetic biology efforts to improve photosynthetic carbon assimilation by introducing carbon concentration mechanisms (e.g. Chen *et al*., 2023; Croce *et al*., 2024), engineering C_4_ metabolism in C_3_ crops (e.g. Schlüter & Weber, 2020; Ermakova *et al*., 2021; Croce *et al*., 2024), introducing novel pathways of carbon assimilation (e.g. Bernhardsgrütter *et al*., 2021; Schulz‐Mirbach *et al*., 2024) and improving the performance of naturally evolved photosynthetic reactions (Smith *et al*., 2023; Croce *et al*., 2024).

## Competing interests

None declared.

## Author contributions

JK, MH, and LW designed the study. LW conducted standard validation experiments. LW and YR conducted the dynamic ^13^CO_2_ labeling experiments. LW and AE analyzed the data. LW and JK wrote the manuscript. All authors edited the manuscript and approved the final version of the manuscript.

## Disclaimer

The New Phytologist Foundation remains neutral with regard to jurisdictional claims in maps and in any institutional affiliations.

## Supporting information


**Fig. S1** Detection limit of oxaloacetate analyzed by GC‐EI‐MS.
**Fig. S2** Gas chromatographic separation of aspartic acid TMS derivatives by GC‐EI‐MS and GC‐APCI‐MS.
**Fig. S3** EI‐induced fragmentation of 3TMS‐derivatized aspartic acid.
**Fig. S4** EI‐ and APCI‐induced fragmentation of aspartic acid 2TMS.
**Fig. S5** EI‐ and APCI‐induced fragmentation of aspartic acid 3TBDMS.
**Fig. S6** In silico fragmentation analysis of aspartic acid 3TMS.
**Fig. S7** Accuracy and precision of E^13^C determination of aspartic acid 3TMS fragments analyzed by GC‐EI‐MS.
**Fig. S8** Accuracy and precision of E^13^C determination of aspartic acid 2TMS fragments and adducts analyzed by GC‐APCI‐MS.
**Fig. S9** Accuracy and precision of E^13^C determination of aspartic acid 3TBDMS fragments and adducts analyzed by GC‐APCI‐MS.
**Fig. S10** Positional E^13^C calculations of aspartic acid using aspartic acid 3TMS and 2TMS analyzed by GC‐APCI‐MS.
**Fig. S11** Positional E^13^C calculations of aspartic acid using aspartic acid 3TMS analyzed by GC‐EI‐MS.
**Fig. S12** Positional E^13^C calculations of aspartic acid using aspartic acid 3TBDMS and 2TBDMS analyzed by GC‐APCI‐MS.
**Fig. S13** Sigmoidal curve fitting of 1‐C and 4‐C from aspartate after the dynamic labeling of *Synechocystis* cultures during the day and the night.
**Table S1** Composition of standard mixtures.
**Table S2** Fragment ion validation of trimethylsilylated and *tert*.‐butyldimethylsilylated derivatives of aspartic acid.
Please note: Wiley is not responsible for the content or functionality of any Supporting Information supplied by the authors. Any queries (other than missing material) should be directed to the *New Phytologist* Central Office.

## Data Availability

The data supporting the findings of this study are presented in Figs 1, 2, 3, 4, S1–S13, and Tables S1, S2 of this article.
