# Supplementary material for: Positional 13C enrichment analysis of aspartate determines PEPC activity in vivo
Source: New Phytol. 2025 Jul 24;248(1):401–14. doi: 10.1111/nph.70412 (PMC12409097; doi:10.1111/nph.70412)
Supplement: Supplementary file 1 — Fig. S1 Detection limit of oxaloacetate analyzed by GC‐EI‐MS. Fig. S2 Gas chromatographic separation of aspartic acid TMS derivatives by GC‐EI‐MS and GC‐APCI‐MS. Fig. S3 EI‐induced fragmentation of 3TMS‐derivatized aspartic acid. Fig. S4 EI‐ and APCI‐induced fragmentation of aspartic acid 2TMS. Fig. S5 EI‐ and APCI‐induced fragmentation of aspartic acid 3TBDMS. Fig. S6 In silico fragmentation analysis of aspartic acid 3TMS. Fig. S7 Accuracy and precision of E13C determination of aspartic acid 3TMS fragments analyzed by GC‐EI‐MS. Fig. S8 Accuracy and precision of E13C determination of aspartic acid 2TMS fragments and adducts analyzed by GC‐APCI‐MS. Fig. S9 Accuracy and precision of E13C determination of aspartic acid 3TBDMS fragments and adducts analyzed by GC‐APCI‐MS. Fig. S10 Positional E13C calculations of aspartic acid using aspartic acid 3TMS and 2TMS analyzed by GC‐APCI‐MS. Fig. S11 Positional E13C calculations of aspartic acid using aspartic acid 3TMS analyzed by GC‐EI‐MS. Fig. S12 Positional E13C calculations of aspartic acid using aspartic acid 3TBDMS and 2TBDMS analyzed by GC‐APCI‐MS. Fig. S13 Sigmoidal curve fitting of 1‐C and 4‐C from aspartate after the dynamic labeling of Synechocystis cultures during the day and the night. Table S1 Composition of standard mixtures. Table S2 Fragment ion validation of trimethylsilylated and tert.‐butyldimethylsilylated derivatives of aspartic acid. Please note: Wiley is not responsible for the content or functionality of any Supporting Information supplied by the authors. Any queries (other than missing material) should be directed to the New Phytologist Central Office. [file NPH-248-401-s001.pdf]

New Phytologist

***New Phytologist* Supporting Information**

Article title: Positional ^{13}C Enrichment Analysis of Aspartate Determines PEPC Activity *In Vivo*
Authors: Luisa Wittemeier, Yogeswari Rajarathinam, Alexander Erban, Martin Hagemann, Joachim Kopka
Article acceptance date: 29 June 2025

The following Supporting Information is available for this article:

Fig. S1 Detection limit of oxaloacetate analyzed by GC-EI-MS.

Fig. S2 Gas chromatographic separation of aspartic acid TMS derivatives by GC-EI-MS and GC-APCI-MS.

Fig. S3 EI-induced fragmentation of aspartic acid 3TMS.

Fig. S4 EI- and APCI-induced fragmentation of aspartic acid 2TMS.

Fig. S5 EI- and APCI-induced fragmentation of aspartic acid 3TBDMS.

Fig. S6 In silico fragmentation analysis of aspartic acid 3TMS.

Fig. S7 Accuracy and precision of $E^{13}C$ determination of aspartic acid 3TMS fragments analyzed by GC-EI-MS.

Fig. S8 Accuracy and precision of $E^{13}C$ determination of aspartic acid 2TMS fragments and adducts analyzed by GC-APCI-MS.

Fig. S9 Accuracy and precision of $E^{13}C$ determination of aspartic acid 3TBDMS fragments and adducts analyzed by GC-APCI-MS.

Fig. S10 Positional $E^{13}C$ calculations of aspartic acid using aspartic acid 3TMS and 2TMS analyzed by GC-APCI-MS.

Fig. S11 Positional $E^{13}C$ calculations of aspartic acid using aspartic acid 3TMS analyzed by GC-EI-MS.

Fig. S12 Positional $E^{13}C$ calculations of aspartic acid using aspartic acid 3TBDMS and 2TBDMS analyzed by GC-APCI-MS.

Fig. S13 Sigmoidal curve fitting of aspartate 1-C and 4-C labeling within *Synechocystis* cultures during the day and the night.

Table S1 Composition of aspartic acid standard mixtures.

Table S2 Fragment ion validation of trimethylsilylated and *tert.*-butyldimethylsilylated derivatives of aspartic acid.

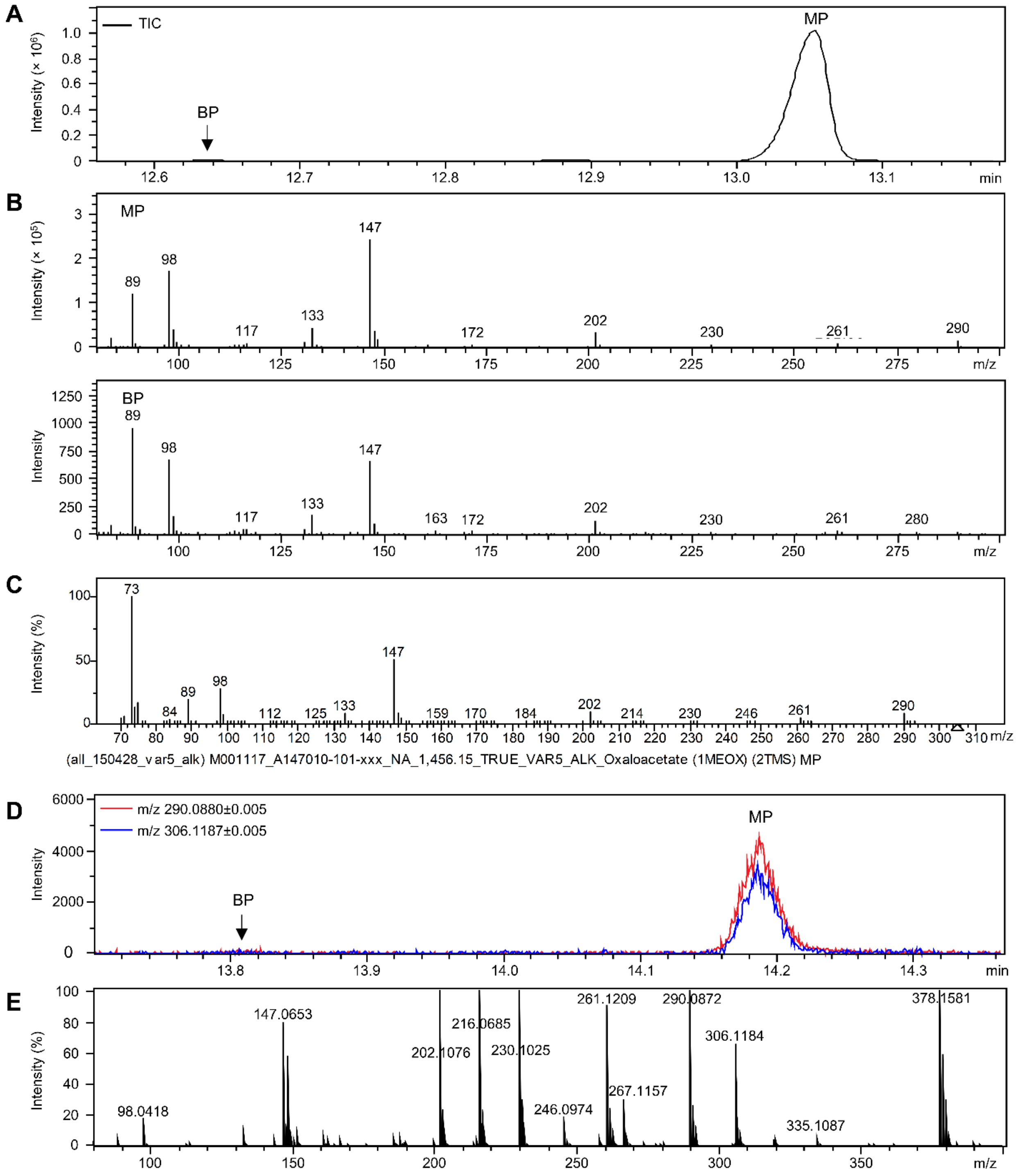

Fig. S1 Detection limit of oxaloacetate analyzed by GC-EI-MS. (A, B) Oxaloacetate was methoxyaminated and trimethylsilylated. 30 ng were injected and analyzed by GC-EI-MS. The analytes Oxaloacetate 1MEOX 2TMS MP (A147010-101, main product) and Oxaloacetate 1MEOX 2TMS BP (A142008-101, by-product), indicated by a vertical arrow, were detected in

the chromatographic retention time range 12.5-13.2 min, total ion chromatogram (TIC) (A). Retention indices (RIs) based on n-alkanes were 1,454.46 and 1,426.15 and matched to the reference RIs, 1,456.15 and 1,427.93, respectively, of the Golm Metabolome Database (GMD); http://gmd.mpimp-golm.mpg.de/. No peaks were detected when less than 30 ng were injected. (B) Characteristic mass spectra of Oxaloacetate 1MEOX 2TMS MP (top) and BP (bottom). (C) Mass spectrum of Oxaloacetate 1MEOX 2TMS MP from the GMD database. (D) Oxaloacetate was methoxyaminated and trimethylsilylated, and 1 ng analyzed by GC-APCI-MS. Characteristic mass features are m/z 306.1187, i.e. the H^+ adduct $[M+H]^+$, and m/z 290.0880, i.e. CH_4 elimination product from the H^+ adduct $[M+H - CH4]^+$. Oxaloacetate 1MEOX 2TMS MP and BP (indicated by vertical arrow) were detected. No peaks were observed by GC-APCI-MS when less than 1 ng was injected for GC-APCI-MS analyses. (E) Mass spectrum of Oxaloacetate 1MEOX 2TMS MP measured by GC-APCI-MS using an injected amount of 5 ng.

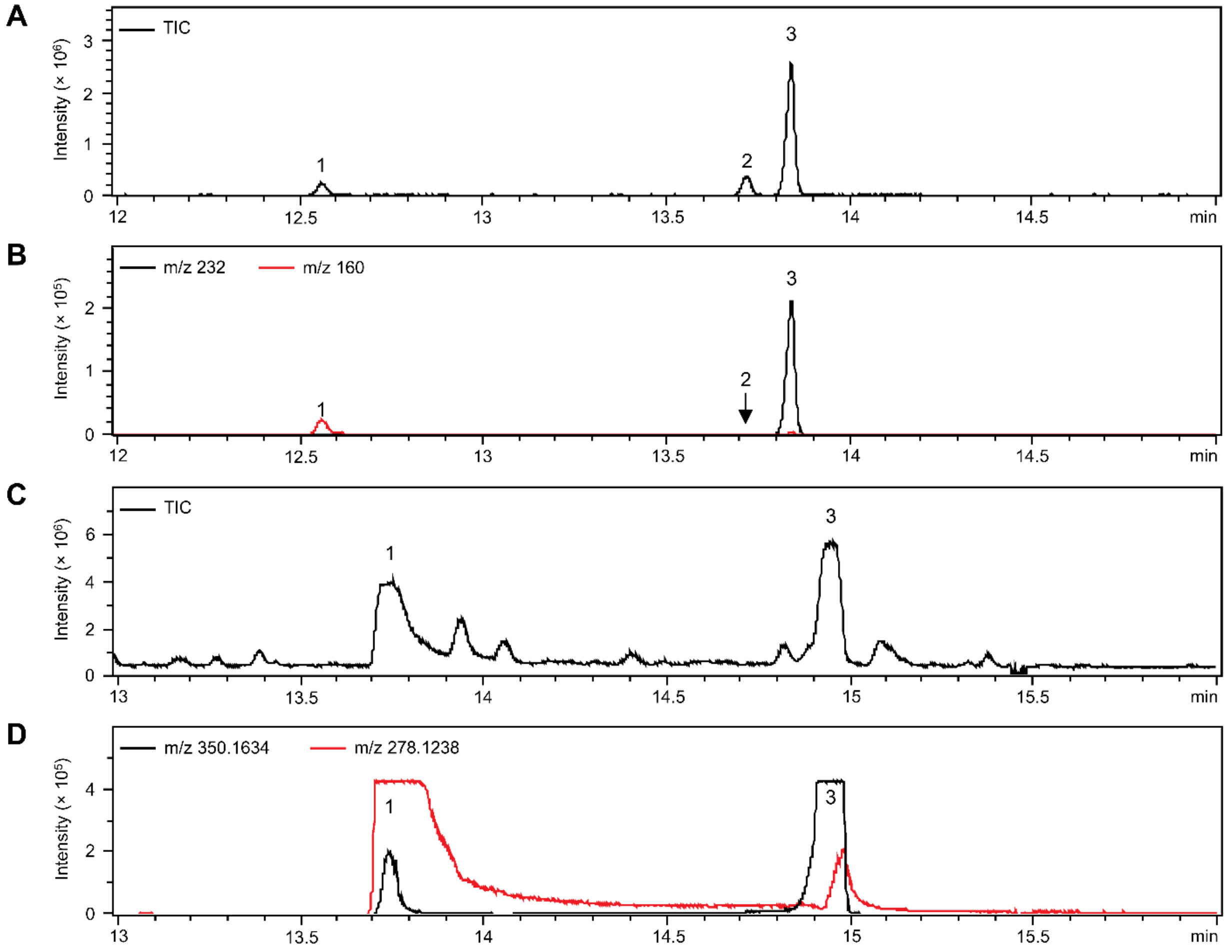

Fig. S2 Gas chromatographic separation of aspartic acid TMS derivatives by GC-EI-MS and GC-APCI-MS. Natural aspartic acid was trimethylsilylated (TMS) and 25 ng of aspartic acid subjected to GC-EI-MS (A, B) or GC-APCI-MS (C, D). Two aspartic acid analytes are detectable by both instruments, aspartic acid 2TMS (peak 1) and aspartic acid 3TMS (peak 3) (A, C). Peak 2 indicated within the EI total ion chromatograms (TIC) represents *n*-pentadecane that is used for retention index standardization. The position of *n*-pentadecane is indicated by an arrow within the extracted ion chromatograms representing aspartic acid 2TMS (m/z 160) and aspartic acid 3TMS (m/z 232) (B). TIC of aspartic acid 2TMS (peak 1) and aspartic acid 3TMS (peak 3) analyzed by GC-APCI-MS (C). Characteristic mass features from GC-APCI-MS analyses are aspartic acid 2TMS (m/z 278.1238) and aspartic acid 3TMS (m/z 350.1634) (D). Note that aspartic acid 2TMS and 3TMS are overloaded (C, D) at 25 ng aspartic acid injected. This analysis demonstrates differences of instrument sensitivity. Different elution times of GC-EI-MS and GC-APCI-MS are

caused by different temperature limits of the gas chromatographic transfer units and the different ionization modes of the mass spectrometers. In case of GC-EI-MS, the capillary GC column ends in a high vacuum for electron impact ionization, in case of GC-APCI-MS, the GC effluent is transferred into an atmospheric pressure environment.

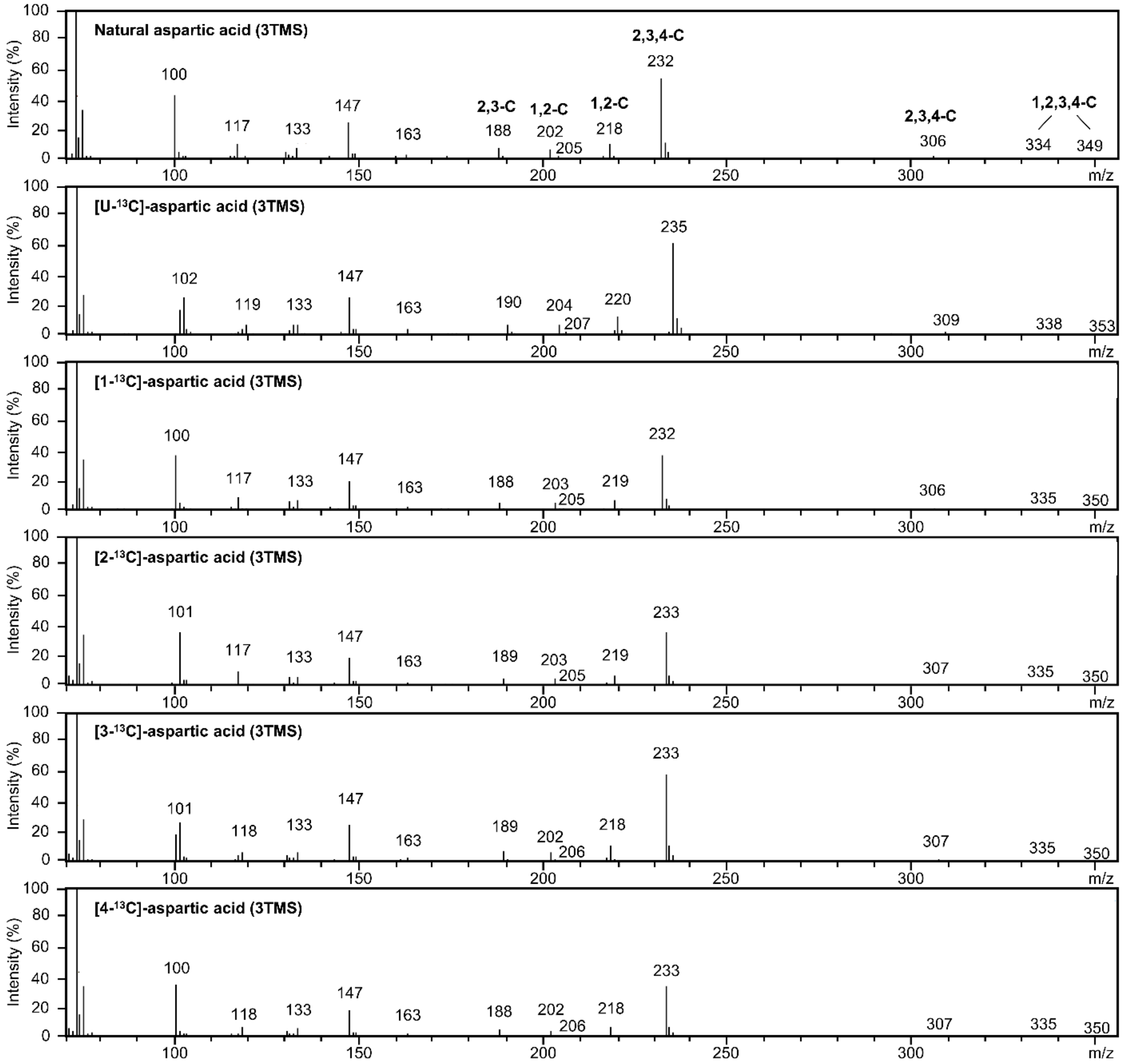

Fig. S3 EI-induced fragmentation of aspartic acid 3TMS. Natural, fully, and position-specific ^{13}C-labeled aspartic acids were trimethylsilylated (TMS) and analyzed by gas-chromatography (GC) coupled to electron ionization (EI) - mass spectrometry (MS). Representative mass spectra of the aspartic acid 3TMS derivatives are displayed. Mass shifts of fragment ions from fully labeled [U-^{13}C]-aspartic acid indicate the numbers of included carbon atoms originating from aspartic acid. Mass shifts of fragment ions from positional labeled aspartic acids provide C-positional information of the C-atoms present within each fragment ion. Fragment interpretations are indicated within the mass spectrum of natural aspartic acid (top). All fragment abundances are intensities (%) normalized to the base peak abundance of each mass spectrum.

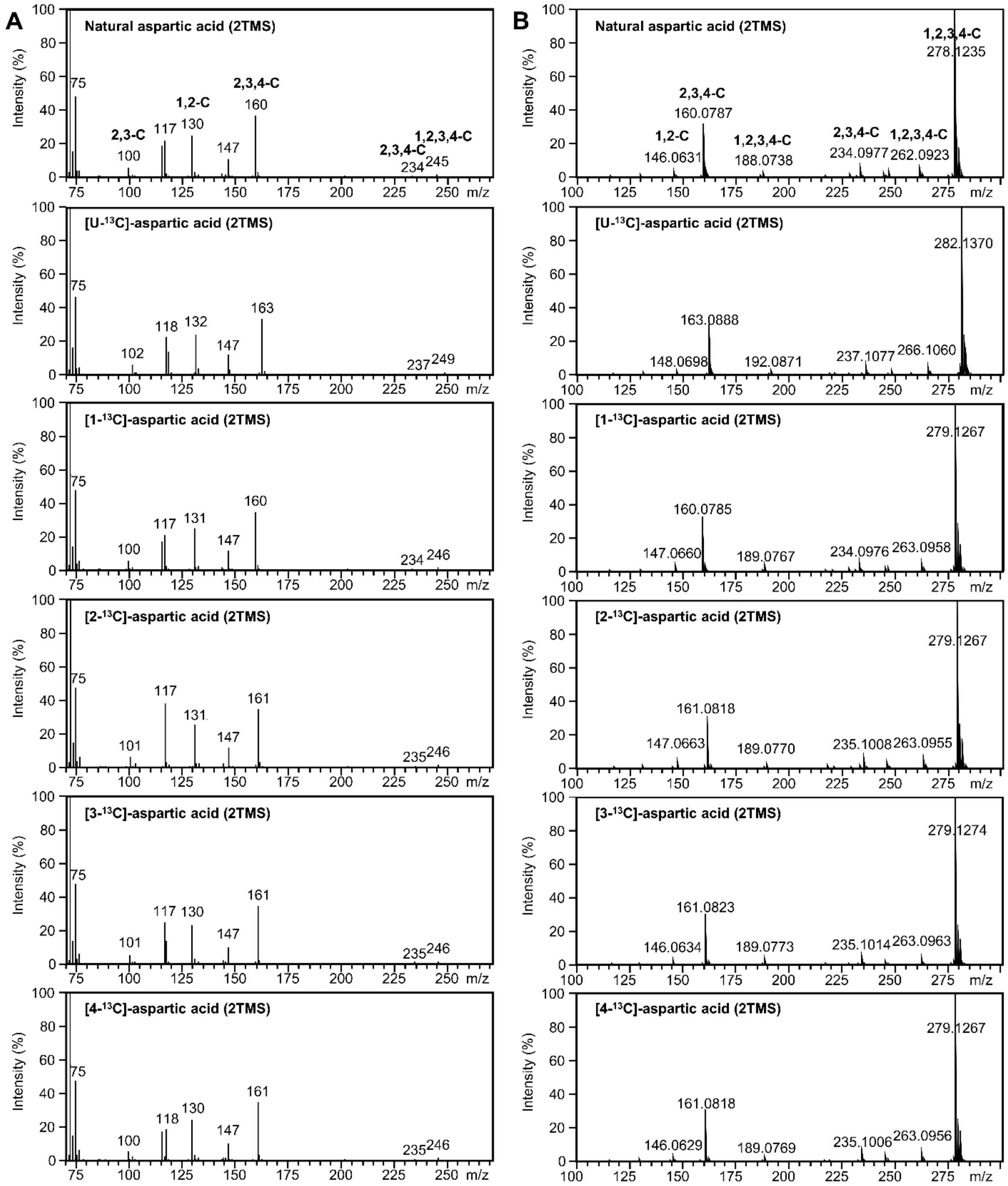

Fig. S4 EI- and APCI-induced fragmentation of aspartic acid 2TMS. Natural, fully, and position-specific ^{13}C-labeled aspartic acids were trimethylsilylated (TMS) and analyzed by gas-chromatography (GC) coupled to electron ionization (EI) (A) or atmospheric pressure chemical

ionization (APCI) mass spectrometry (MS) (B). Representative mass spectra of the aspartic acid 2TMS derivatives are displayed. Mass shifts of fragment ions from fully labeled [U-^{13}C]-aspartic acid indicate the numbers of included carbon atoms originating from aspartic acid. Mass shifts of fragment ions from positional labeled aspartic acids provide C-positional information of the C-atoms present within each fragment ion. Fragment interpretations are indicated within the mass spectrum of natural aspartic acid (top). All fragment abundances are intensities (%) normalized to the base peak abundance of each mass spectrum.

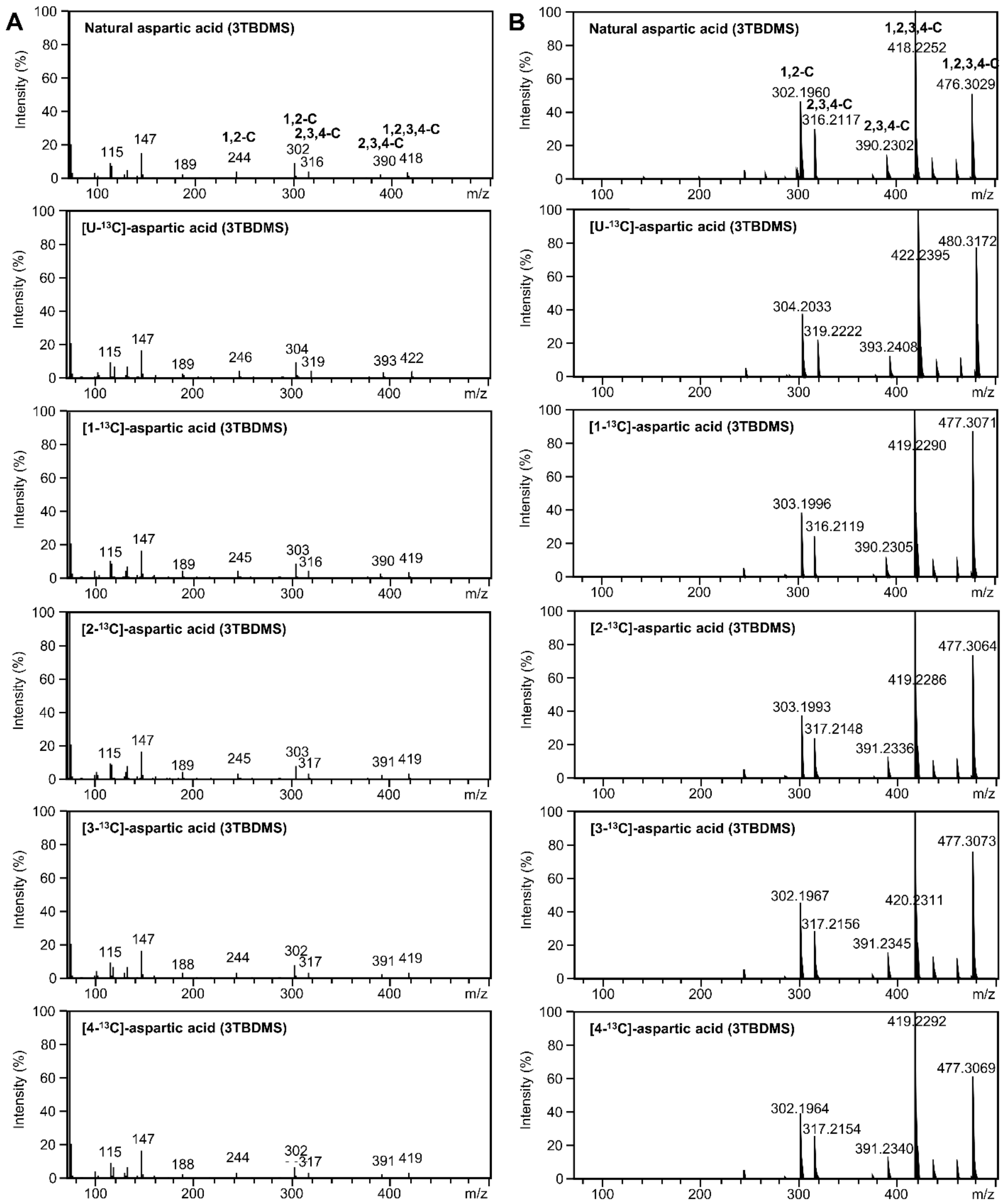

Fig. S5 EI- and APCI-induced fragmentation of aspartic acid 3TBDMS. Natural, fully, and position-specific ^{13}C-labeled aspartic acids were *tert.*-butyldimethylsilylated (TBDMS) and analyzed by gas-chromatography (GC) coupled to electron ionization (EI; A) or atmospheric pressure chemical ionization (APCI) mass spectrometry (MS; B). Representative mass spectra of

the aspartic acid 3TBDMS derivatives are displayed. Mass shifts of fragment ions from fully labeled [U-^{13}C]-aspartic acid indicate the numbers of included carbon atoms originating from aspartic acid. Mass shifts of fragment ions from positional labeled aspartic acids provide C-positional information of the C-atoms present within each fragment ion. Fragment interpretations are indicated within the mass spectrum of natural aspartic acid (top). All fragment abundances are intensities (%) normalized to the base peak abundance of each mass spectrum.

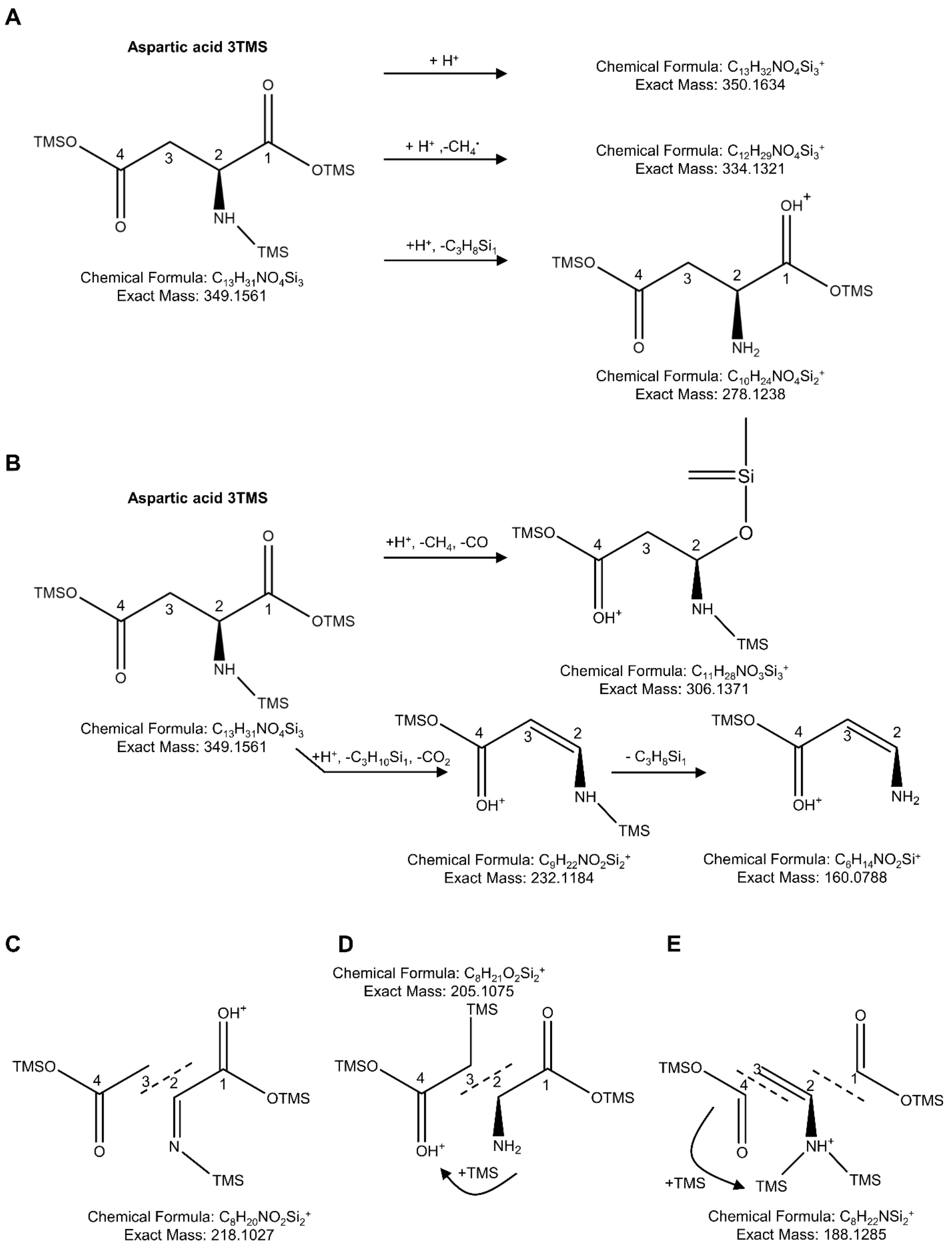
A
Aspartic acid 3TMS
+ H+
Chemical Formula: C13H32NO4Si3+
Exact Mass: 350.1634
+ H+ ,-CH4·
Chemical Formula: C12H29NO4Si3+
Exact Mass: 334.1321
+H+, -C3H8Si1
Chemical Formula: C13H31NO4Si3
Exact Mass: 349.1561
Chemical Formula: C10H24NO4Si2+
Exact Mass: 278.1238
B
Aspartic acid 3TMS
+H+, -CH4, -CO
Chemical Formula: C11H28NO3Si3+
Exact Mass: 306.1371
Chemical Formula: C13H31NO4Si3
Exact Mass: 349.1561
+H+, -C3H10Si1, -CO2
- C3H8Si1
Chemical Formula: C9H22NO2Si2+
Exact Mass: 232.1184
Chemical Formula: C6H14NO2Si+
Exact Mass: 160.0788
C
Chemical Formula: C8H20NO2Si2+
Exact Mass: 218.1027
D
Chemical Formula: C8H21O2Si2+
Exact Mass: 205.1075
+TMS
E
+TMS
Chemical Formula: C8H22NSi2+
Exact Mass: 188.1285

Fig. S6 *In silico* fragmentation analysis of aspartic acid 3TMS. Aspartic acid 3TMS fragmentation by GC-APCI-MS was predicted through *in silico* analyses assuming initial protonation, $[M+H]^+$, and sequential neutral elimination, intramolecular rearrangement or intermolecular transfer reactions. (A) Different fragment ions with the complete carbon backbone of aspartic acid (1,2,3,4-C) are formed through eliminations of methyl- and/or TMS-groups after proton adduct formation. These eliminated moieties originate from the chemical derivatization reagent. (B) Fragment ions including 2,3,4-C of the aspartic acid carbon-backbone are interpreted as result of cleavage between 1-C and 2-C or eliminations of CO or CO_2 with rearrangements and progressive eliminations of the TMS-moieties. (C) Fragment ion m/z 218 (1,2-C) is thought to arise from cleavage of the aspartic acid molecule between 2-C and 3-C. (D) Likely, rearrangement or transfer of one TMS group to the 3,4-C fragment leads to the formation of fragment ion m/z 205. (E) Cleavages between 1-C/ 2-C and 3-C/ 4-C with rearrangement or transfer of one TMS group to the amino group can explain fragment ion m/z 188 (2,3-C). Suggested structures are exemplary and may represent one of multiple possible isomers.

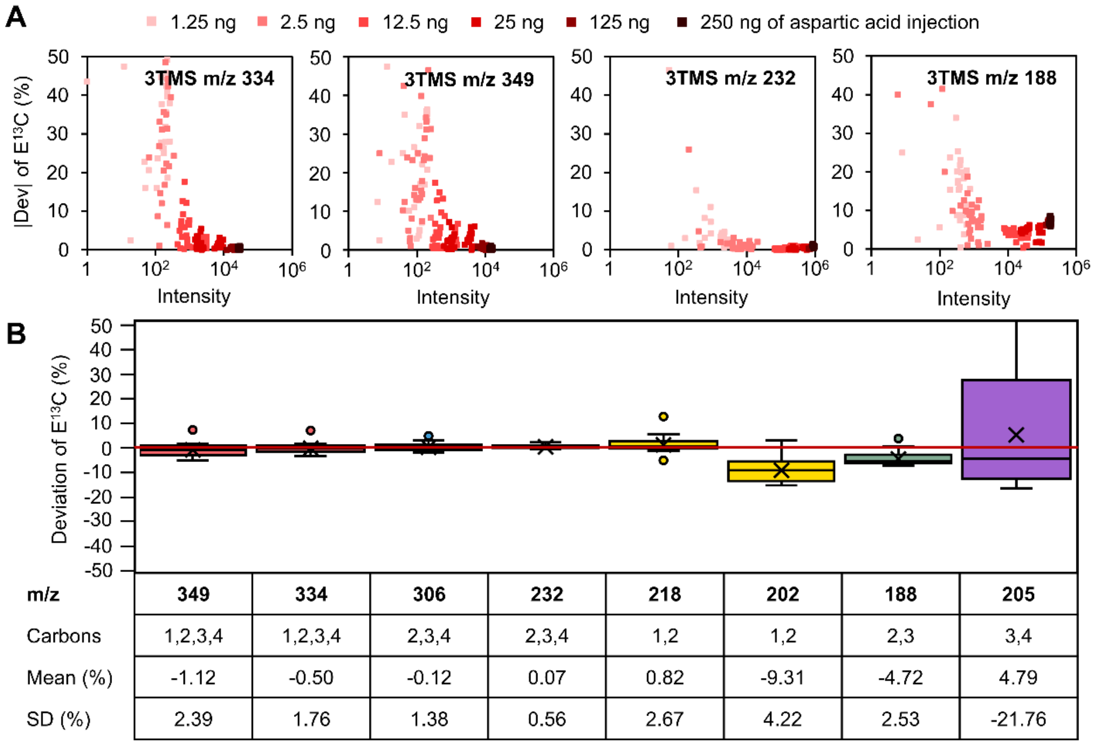

m/z	349	334	306	232	218	202	188	205
Carbons	1,2,3,4	1,2,3,4	2,3,4	2,3,4	1,2	1,2	2,3	3,4
Mean (%)	-1.12	-0.50	-0.12	0.07	0.82	-9.31	-4.72	4.79
SD (%)	2.39	1.76	1.38	0.56	2.67	4.22	2.53	-21.76

Fig. S7 Accuracy and precision of fractional ^{13}C enrichment ($E^{13}C$) determination of aspartic acid 3TMS fragments analyzed by GC-EI-MS. Equal mixtures of four positional ^{13}C-labeled aspartic acid standards in different isotopic dilutions with natural aspartic acid and at different concentrations (Table S1) were measured by GC-EI-MS. Deviations of $E^{13}C$, i.e., measured $E^{13}C$ subtracted from expected $E^{13}C$ (|Dev|), of the specified fragment were analyzed. (A) Absolute values of $E^{13}C$ deviations depend on the specific abundances, i.e., the sum of all isotopologue abundances, of the selected fragments and on the amounts of injected aspartic acid. Most fragments provide accurate enrichment information with injections ≥ 125 ng aspartic acid. Fragment m/z 232 has high $E^{13}C$ accuracy for injections higher 12.5 ng. Fragment m/z 188 shows absolute deviation of $E^{13}C$ of about 5% also at 250 ng injected. (B) Box-plot representation of $E^{13}C$ deviations with means, and standard deviations (SD) of selected fragments. Fragments are defined by nominal mass to charge ratio (m/z) and included carbon-atoms of aspartic acid (left to right: red, 1,2,3,4-C; blue, 2,3,4-C; yellow, 1,2-C; green, 2,3-C; purple, 3,4-C). Thirty-six different mixtures were analyzed by 4 technical replicates each at 25 ng aspartic acid injected. The mean deviations and standard deviations (SD) provide accuracy and precision information, respectively. All fragments were analyzed by GC-EI-MS

measurements in split-less mode. (Standard box plot elements: x, average; boxes, 75th percentile, median, and 25th percentile; whiskers, 100th percentile and 0th percentile; outliers, circles).

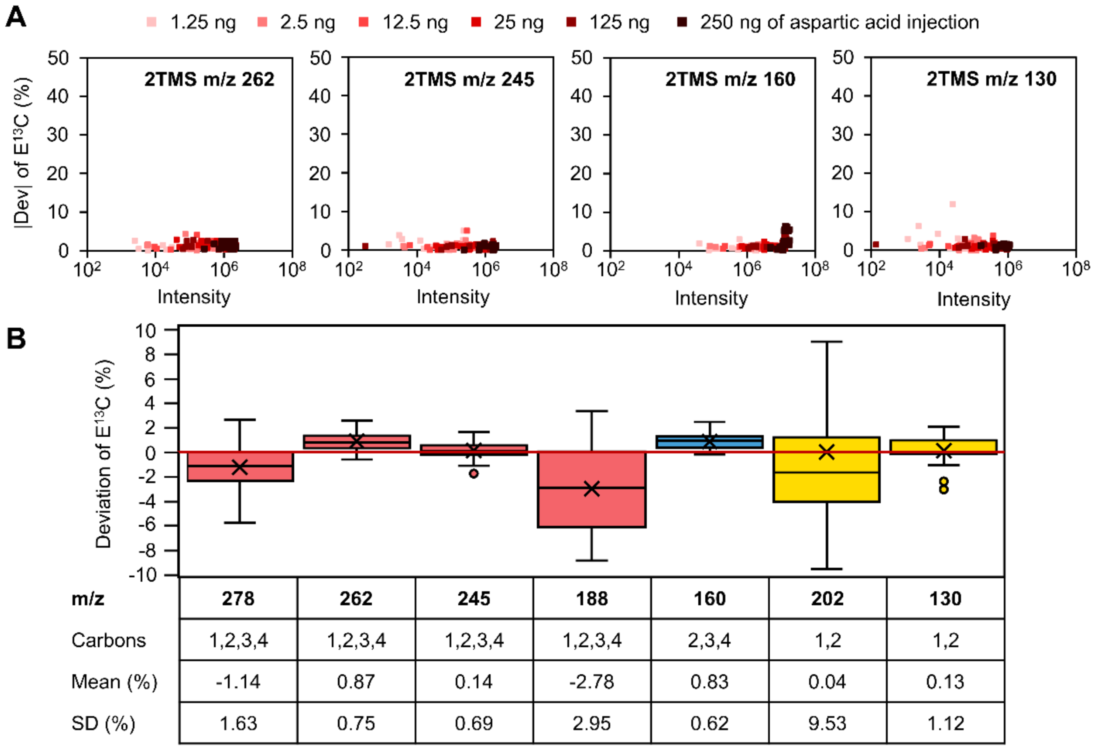

m/z	278	262	245	188	160	202	130
Carbons	1,2,3,4	1,2,3,4	1,2,3,4	1,2,3,4	2,3,4	1,2	1,2
Mean (%)	-1.14	0.87	0.14	-2.78	0.83	0.04	0.13
SD (%)	1.63	0.75	0.69	2.95	0.62	9.53	1.12

Fig. S8 Accuracy and precision of fractional ^{13}C enrichment ($E^{13}C$) determination of aspartic acid 2TMS fragments and adducts analyzed by GC-APCI-MS. Equal mixtures of four positional ^{13}C-labeled aspartic acid standards in different isotopic dilutions with natural aspartic acid and at different concentrations (Table S1) were measured by GC-APCI-MS. Deviations of $E^{13}C$, i.e., measured $E^{13}C$ subtracted from expected $E^{13}C$, of the specified fragment were analyzed. (A) Absolute values of $E^{13}C$ deviations depend on the specific abundances, i.e., the sum of all isotopologue abundances, of the selected fragments and on the amounts of injected aspartic acid. $E^{13}C$ accuracy of fragments m/z 262, m/z 245, and m/z 130 was mostly independent of fragment intensity. Fragment m/z 160 had reduced $E^{13}C$ accuracy due to saturation at ≥ 125 ng injected. (B) Box-plot representation of $E^{13}C$ deviations with means, and standard deviations (SD) of selected fragments. Fragments are defined by nominal mass to charge ratio (m/z) and included carbon-atoms of aspartic acid (left to right: red, 1,2,3,4-C; blue, 2,3,4-C; yellow, 1,2-C). Thirty-six different mixtures were analyzed by 4 technical replicates each at 25 ng aspartic acid injected. The mean deviations and standard deviations (SD) provide accuracy and precision information, respectively. All fragments were analyzed by GC-APCI-MS measurements in split-

less mode. (Standard box plot elements: x, average; boxes, 75th percentile, median, and 25th percentile; whiskers, 100th percentile and 0th percentile; outliers, circles).

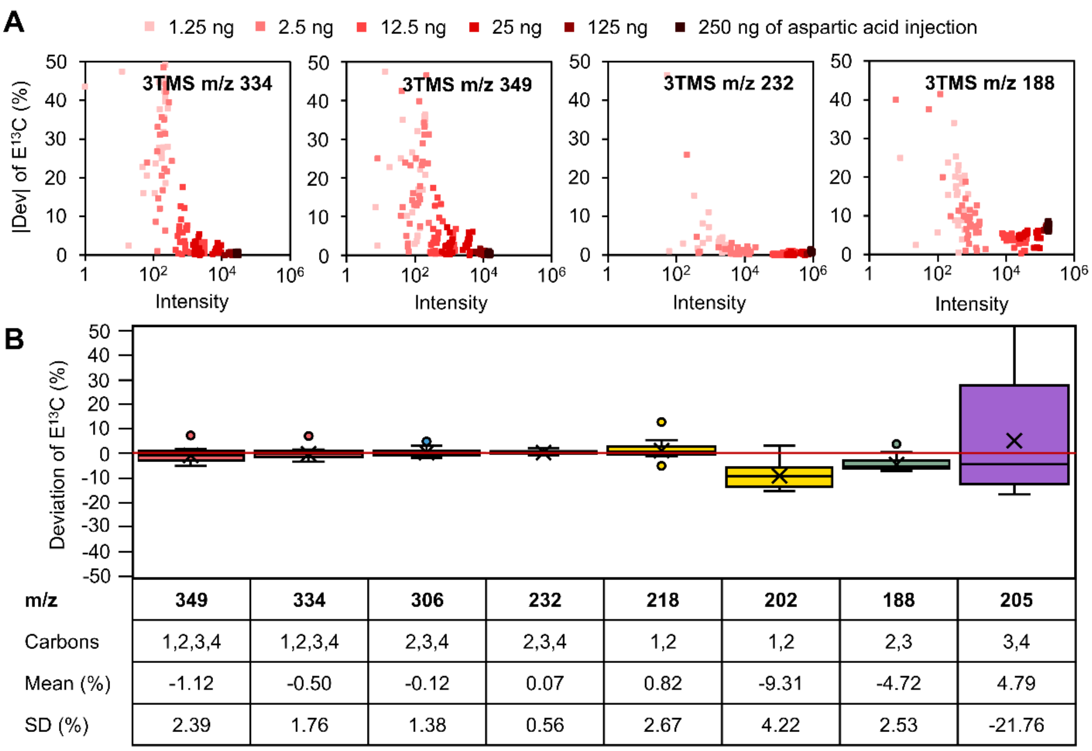

m/z	349	334	306	232	218	202	188	205
Carbons	1,2,3,4	1,2,3,4	2,3,4	2,3,4	1,2	1,2	2,3	3,4
Mean (%)	-1.12	-0.50	-0.12	0.07	0.82	-9.31	-4.72	4.79
SD (%)	2.39	1.76	1.38	0.56	2.67	4.22	2.53	-21.76

Fig. S9 Accuracy and precision of fractional ^{13}C enrichment ($E^{13}C$) determination of aspartic acid 3TBDMS fragments and adducts analyzed by GC-APCI-MS. Equal mixtures of four positional ^{13}C-labeled aspartic acid standards in different isotopic dilutions with natural aspartic acid and at different concentrations (Table S1) were measured by GC-APCI-MS. Deviations of $E^{13}C$, i.e., measured $E^{13}C$ subtracted from expected $E^{13}C$, of the specified fragment were analyzed. (A) Absolute values of $E^{13}C$ deviations depend on the specific abundances, i.e., the sum of all isotopologue abundances, of the selected fragments and on the amounts of injected aspartic acid. Most fragments provide accurate enrichment information with injections ≥ 12.5 ng aspartic acid but saturation is reached in many cases at ≥ 125 ng. (B) Box-plot representation of $E^{13}C$ deviations with means, and standard deviations (SD) of selected fragments. Fragments are defined by nominal mass to charge ratio (m/z) and included carbon-atoms of aspartic acid (left to right: red, 1,2,3,4-C; blue, 2,3,4-C; yellow, 1,2-C; green, 2,3-C; purple, 3,4-C). Thirty-six different mixtures were analyzed by 4 technical replicates each at 25 ng aspartic acid injected. The mean deviations and standard deviations (SD) provide accuracy and precision information, respectively. All fragments were analyzed by GC-APCI-MS measurements in split-less mode.

(Standard box plot elements: x, average; boxes, 75th percentile, median, and 25th percentile; whiskers, 100th percentile and 0th percentile; outliers, circles).

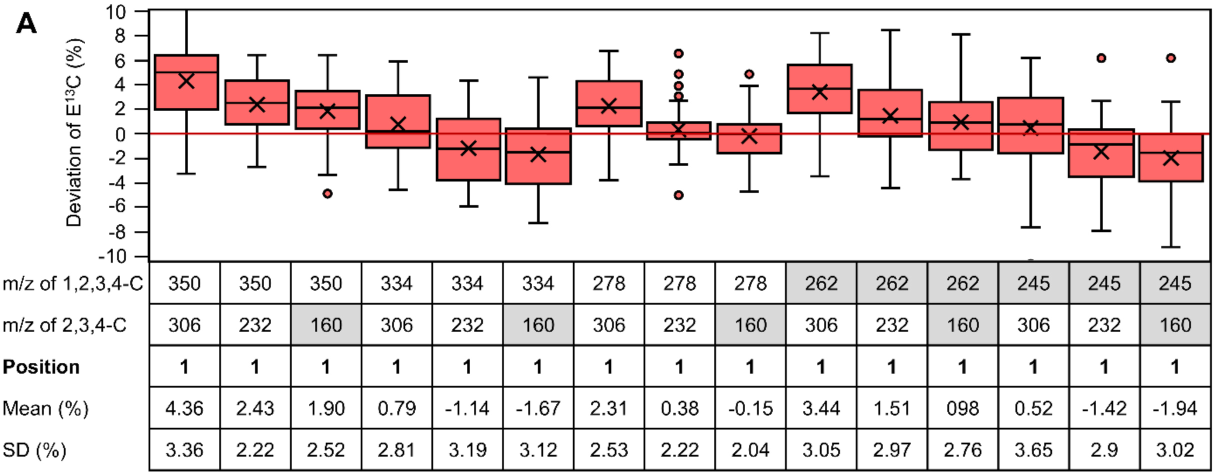

m/z of 1,2,3,4-C	350	350	350	334	334	334	278	278	278	262	262	262	245	245	245
m/z of 2,3,4-C	306	232	160	306	232	160	306	232	160	306	232	160	306	232	160
**Position**	**1**	**1**	**1**	**1**	**1**	**1**	**1**	**1**	**1**	**1**	**1**	**1**	**1**	**1**	**1**
Mean (%)	4.36	2.43	1.90	0.79	-1.14	-1.67	2.31	0.38	-0.15	3.44	1.51	098	0.52	-1.42	-1.94
SD (%)	3.36	2.22	2.52	2.81	3.19	3.12	2.53	2.22	2.04	3.05	2.97	2.76	3.65	2.9	3.02

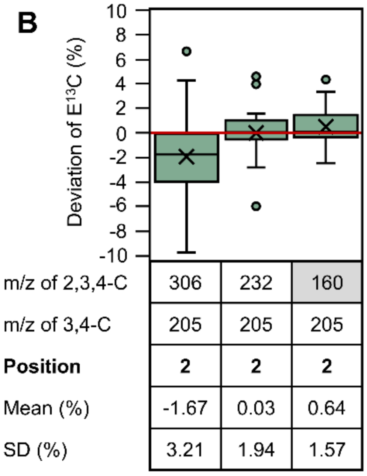

m/z of 2,3,4-C	306	232	160
m/z of 3,4-C	205	205	205
**Position**	**2**	**2**	**2**
Mean (%)	-1.67	0.03	0.64
SD (%)	3.21	1.94	1.57

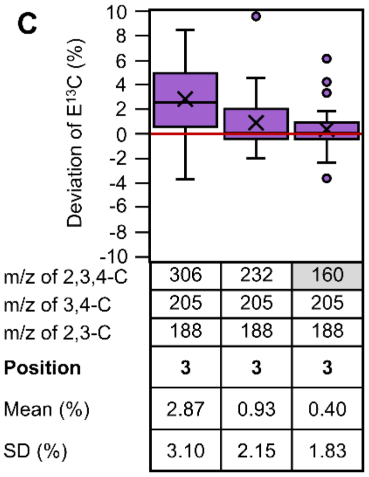

m/z of 2,3,4-C	306	232	160
m/z of 3,4-C	205	205	205
m/z of 2,3-C	188	188	188
**Position**	**3**	**3**	**3**
Mean (%)	2.87	0.93	0.40
SD (%)	3.10	2.15	1.83

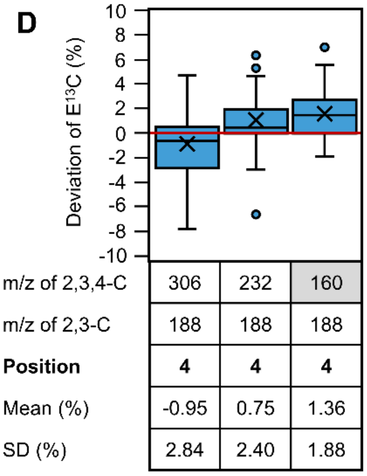

m/z of 2,3,4-C	306	232	160
m/z of 2,3-C	188	188	188
**Position**	**4**	**4**	**4**
Mean (%)	-0.95	0.75	1.36
SD (%)	2.84	2.40	1.88

Fig. S10 Positional fractional ^{13}C enrichment ($E^{13}C$) calculations of aspartic acid using aspartic acid 3TMS and 2TMS analyzed by GC-APCI-MS.Positional $E^{13}C$ was calculated by equations (1), (2), (3) and (4) using $E^{13}C$ of the indicated fragments (m/z, grey underlay indicates fragments from aspartic acid 2TMS, white from aspartic acid 3TMS). Distribution analyses of $E^{13}C$ deviations, i.e., calculated $E^{13}C$ subtracted from expected, is displayed across 36 different mixtures with 4 technical replicates. Amount of total aspartic acid injected was 25 ng. $E^{13}C$ of fragment m/z 232 was determined by split measurements at split ratio 1:5. $E^{13}Cs$ of all other fragments were determined in split-less mode. (A) 1-C of aspartic acid (red), (B) 2-C of aspartic acid (green), (C) 3-C of aspartic acid (purple), (D) 4-C of aspartic acid (blue). The mean deviations and standard deviations (SD) provide accuracy and precision information, respectively. (Standard box plot elements: x, average; boxes, 75th percentile, median, and 25th percentile; whiskers, 100th percentile and 0th percentile; outliers, circles).

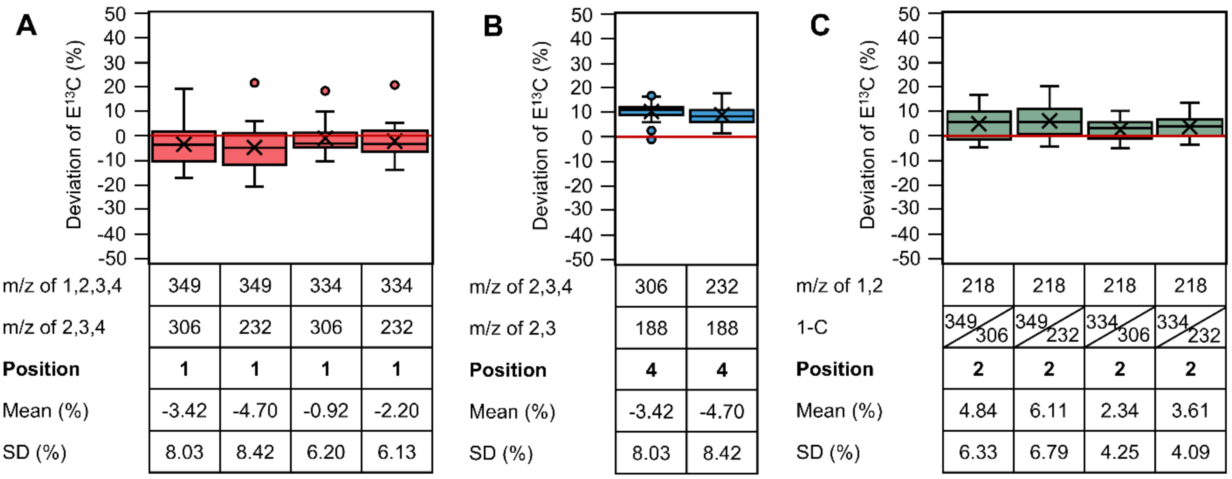

A				
m/z of 1,2,3,4	349	349	334	334
m/z of 2,3,4	306	232	306	232
**Position**	**1**	**1**	**1**	**1**
Mean (%)	-3.42	-4.70	-0.92	-2.20
SD (%)	8.03	8.42	6.20	6.13

B		
m/z of 2,3,4	306	232
m/z of 2,3	188	188
**Position**	**4**	**4**
Mean (%)	-3.42	-4.70
SD (%)	8.03	8.42

C				
m/z of 1,2	218	218	218	218
1-C	349/306	349/232	334/306	334/232
**Position**	**2**	**2**	**2**	**2**
Mean (%)	4.84	6.11	2.34	3.61
SD (%)	6.33	6.79	4.25	4.09

Fig. S11 Positional fractional ^{13}C enrichment ($E^{13}C$) calculations of aspartic acid using aspartic acid 3TMS analyzed by GC-EI-MS.Positional $E^{13}C$ was calculated by equations (1) and (4) using $E^{13}C$ of the indicated fragments (m/z). $E^{13}C$ of 2-C (C) was determined by $E^{13}C$ of 1-C (A) and $E^{13}C$ of fragment m/z 218 that included 1,2-C. Distribution analyses of $E^{13}C$ deviations, i.e., calculated $E^{13}C$ subtracted from expected, is displayed across 36 different mixtures with 4 technical replicates. Amount of total aspartic acid injected was 25 ng. $E^{13}Cs$ of all fragments were determined in split-less mode. Positional $E^{13}C$ of 4-C is underestimated likely due to interference of $E^{13}C$ of m/z 188. (A) 1-C of aspartic acid (red), (B) 2-C of aspartic acid (green), (D) 4-C of aspartic acid (blue). The mean deviations and standard deviations (SD) provide accuracy and precision information, respectively. (Standard box plot elements: x, average; boxes, 75th percentile, median, and 25th percentile; whiskers, 100th percentile and 0th percentile; outliers, circles).

A

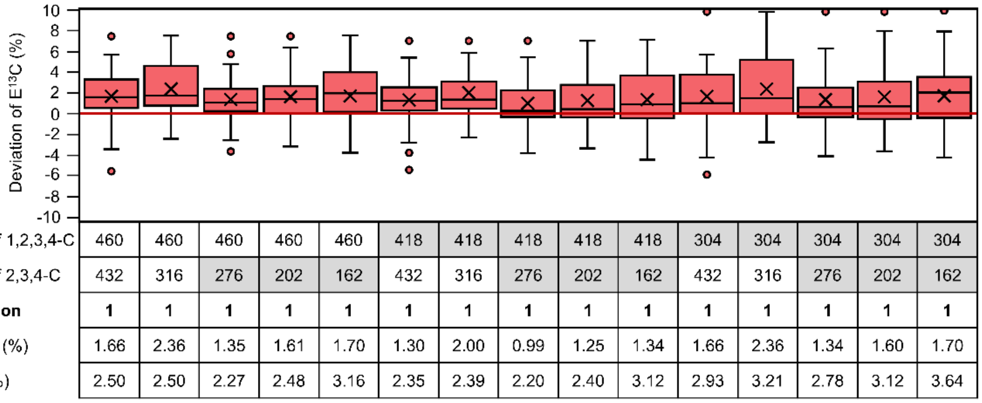

m/z of 1,2,3,4-C	460	460	460	460	460	418	418	418	418	418	304	304	304	304	304
m/z of 2,3,4-C	432	316	276	202	162	432	316	276	202	162	432	316	276	202	162
**Position**	**1**	**1**	**1**	**1**	**1**	**1**	**1**	**1**	**1**	**1**	**1**	**1**	**1**	**1**	**1**
Mean (%)	1.66	2.36	1.35	1.61	1.70	1.30	2.00	0.99	1.25	1.34	1.66	2.36	1.34	1.60	1.70
SD (%)	2.50	2.50	2.27	2.48	3.16	2.35	2.39	2.20	2.40	3.12	2.93	3.21	2.78	3.12	3.64

B

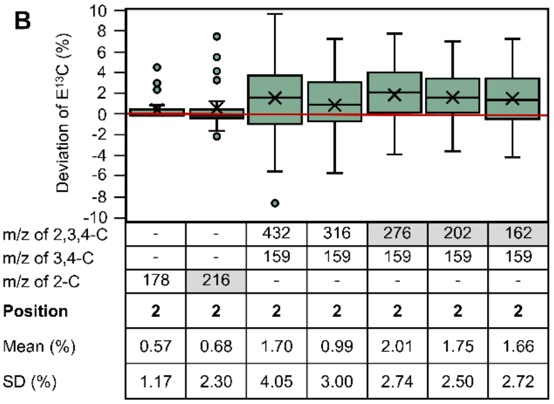

m/z of 2,3,4-C	-	-	432	316	276	202	162
m/z of 3,4-C	-	-	159	159	159	159	159
m/z of 2-C	178	216	-	-	-	-	-
**Position**	**2**	**2**	**2**	**2**	**2**	**2**	**2**
Mean (%)	0.57	0.68	1.70	0.99	2.01	1.75	1.66
SD (%)	1.17	2.30	4.05	3.00	2.74	2.50	2.72

C

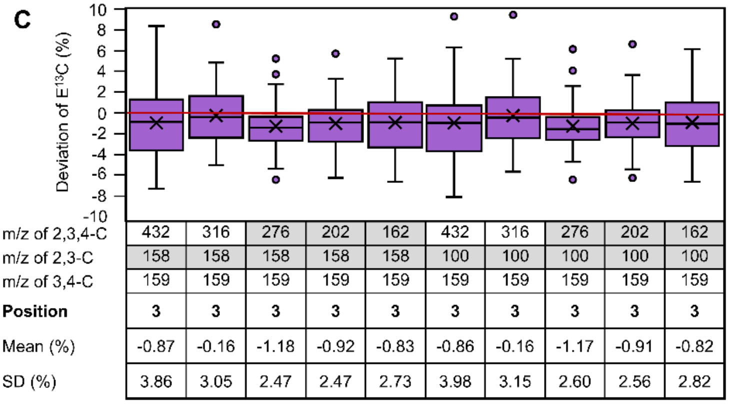

m/z of 2,3,4-C	432	316	276	202	162	432	316	276	202	162
m/z of 2,3-C	158	158	158	158	158	100	100	100	100	100
m/z of 3,4-C	159	159	159	159	159	159	159	159	159	159
**Position**	**3**	**3**	**3**	**3**	**3**	**3**	**3**	**3**	**3**	**3**
Mean (%)	-0.87	-0.16	-1.18	-0.92	-0.83	-0.86	-0.16	-1.17	-0.91	-0.82
SD (%)	3.86	3.05	2.47	2.47	2.73	3.98	3.15	2.60	2.56	2.82

D

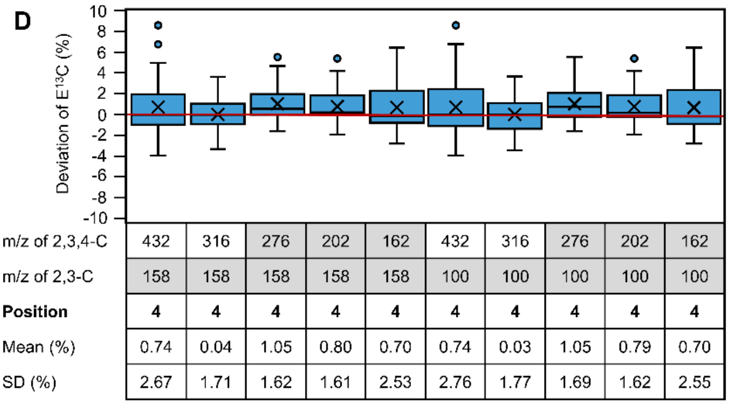

m/z of 2,3,4-C	432	316	276	202	162	432	316	276	202	162
m/z of 2,3-C	158	158	158	158	158	100	100	100	100	100
**Position**	**4**	**4**	**4**	**4**	**4**	**4**	**4**	**4**	**4**	**4**
Mean (%)	0.74	0.04	1.05	0.80	0.70	0.74	0.03	1.05	0.79	0.70
SD (%)	2.67	1.71	1.62	1.61	2.53	2.76	1.77	1.69	1.62	2.55

Fig. S12 Positional fractional ^{13}C enrichment ($E^{13}C$) calculations of aspartic acid using aspartic acid 3TBDMS and 2TBDMS analyzed by GC-APCI-MS. Positional $E^{13}C$ was measured directly (m/z 178 of aspartic acid 3TBDMS and m/z 216 of aspartic acid 2TBDMS) or calculated by equations (1), (2), (3) and (4) using $E^{13}C$ of the indicated fragments (m/z, grey underlay indicates fragments from aspartic acid 2TBDMS, white from aspartic acid 3TBDMS). Distribution analyses of $E^{13}C$ deviations, i.e., calculated $E^{13}C$ subtracted from expected, is displayed across 36 different mixtures with 4 technical replicates. Amount of total aspartic acid injected was 25 ng. $E^{13}Cs$ of all fragments were determined in split-less mode. (A) 1-C of aspartic acid (red), (B) 2-C of aspartic acid (green), (C) 3-C of aspartic acid (purple), (D) 4-C of aspartic acid (blue). The mean deviations and standard deviations (SD) provide accuracy and precision information, respectively. (Standard box plot elements: x, average; boxes, 75th percentile, median, and 25th percentile; whiskers, 100th percentile and 0th percentile; outliers, circles).

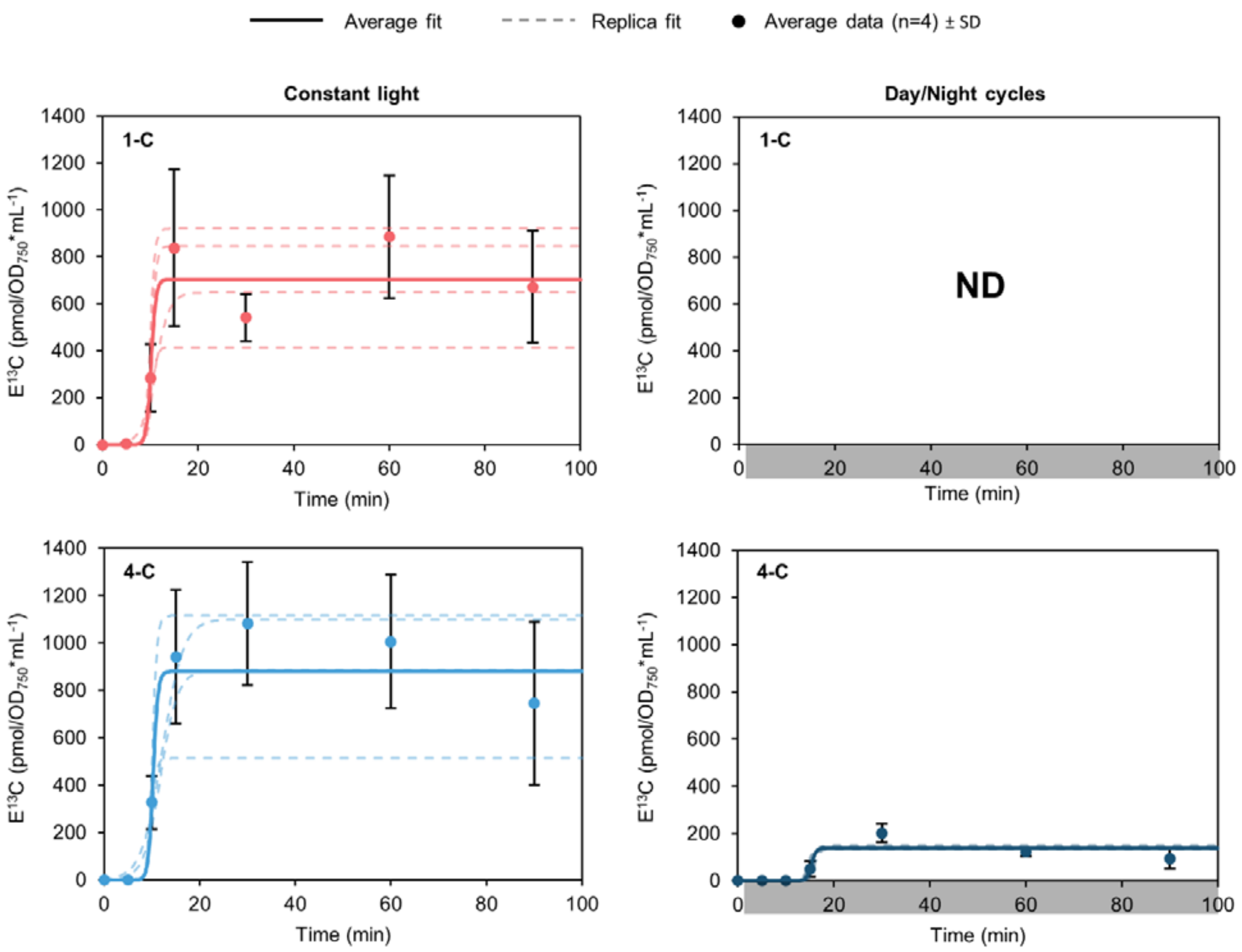

Fig. S13 Sigmoidal curve fitting of aspartate 1-C and 4-C labeling within *Synechocystis* sp. PCC 6803 cultures during the day and the night. *Synechocystis* cells were cultivated photoautotrophically with 5% CO_2-enriched air. Cells were either cultivated in constant light or a 12h:12h day/night photoperiod. For day/night cultivations, the $^{13}CO_2$ labelling pulse was applied directly after transition to the night. Samples were taken 5 to 90 min after the labelling pulse. Samples were analyzed by GC-MS. Data points represent averages of 4 replicates ± SD. Curve fitting was done for molar fractional ^{13}C enrichment ($E^{13}C$) of aspartate 1-C and 4-C to characterize contributions of RUBISCO and PEPC activities, respectively. Sigmoidal fits are displayed of the average data points (solid line) and of the single replicates (n=4, dashed lines). No assimilation of ^{13}C was detected in aspartate at position 1-C during the night (ND = not detected).

Table S1 Composition of aspartic acid standard mixtures. Mixtures to determine the lower threshold of fractional ^{13}C enrichment ($E^{13}C$) detection are shown by green underlay (ID 32-64). Accuracy and precision of $E^{13}C$ were determined by mixtures (yellow underlay; ID 7-31) and ID 42-46 (green underlay) or pure substances (white underlay; ID 1-6) all at 25 ng injected. The amount injected (ng) refers to the amount of aspartic acid standard substance that is injected for GC-MS analyses after chemical derivatization.

		Proportion (%)						Amount injected (ng)					
ID	amount (ng)	natural	U-^{13}C	1-$^{13}C_1$	2-$^{13}C_1$	3-$^{13}C_1$	4-$^{13}C_1$	natural	U-^{13}C	1-$^{13}C_1$	2-$^{13}C_1$	3-$^{13}C_1$	4-$^{13}C_1$
**1**	25	100	0	0	0	0	0	25	0	0	0	0	0
**2**	25	0	100	0	0	0	0	0	25	0	0	0	0
**3**	25	0	0	100	0	0	0	0	0	25	0	0	0
**4**	25	0	0	0	100	0	0	0	0	0	25	0	0
**5**	25	0	0	0	0	100	0	0	0	0	0	25	0
**6**	25	0	0	0	0	0	100	0	0	0	0	0	25
**7**	25	95	0	5	0	0	0	23.75	0	1.25	0	0	0
**8**	25	90	0	10	0	0	0	22.5	0	2.5	0	0	0
**9**	25	50	0	50	0	0	0	12.5	0	12.5	0	0	0
**10**	25	10	0	90	0	0	0	2.5	0	22.5	0	0	0
**11**	25	5	0	95	0	0	0	1.25	0	23.75	0	0	0
**12**	25	95	0	0	5	0	0	23.75	0	0	1.25	0	0
**13**	25	90	0	0	10	0	0	22.5	0	0	2.5	0	0
**14**	25	50	0	0	50	0	0	12.5	0	0	12.5	0	0
**15**	25	10	0	0	90	0	0	2.5	0	0	22.5	0	0
**16**	25	5	0	0	95	0	0	1.25	0	0	23.75	0	0
**17**	25	95	0	0	0	5	0	23.75	0	0	0	1.25	0
**18**	25	90	0	0	0	10	0	22.5	0	0	0	2.5	0
**19**	25	50	0	0	0	50	0	12.5	0	0	0	12.5	0
**20**	25	10	0	0	0	90	0	2.5	0	0	0	22.5	0
**21**	25	5	0	0	0	95	0	1.25	0	0	0	23.75	0
**22**	25	95	0	0	0	0	5	23.75	0	0	0	0	1.25
**23**	25	90	0	0	0	0	10	22.5	0	0	0	0	2.5
**24**	25	50	0	0	0	0	50	12.5	0	0	0	0	12.5
**25**	25	10	0	0	0	0	90	2.5	0	0	0	0	22.5
**26**	25	5	0	0	0	0	95	1.25	0	0	0	0	23.75
**27**	25	0	0	95	0	0	5	0	0	23.75	0	0	1.25
**28**	25	0	0	90	0	0	10	0	0	22.5	0	0	2.5
**29**	25	0	0	50	0	0	50	0	0	12.5	0	0	12.5
**30**	25	0	0	10	0	0	90	0	0	2.5	0	0	22.5
**31**	25	0	0	5	0	0	95	0	0	1.25	0	0	23.75
**32**	250	0	0	25	25	25	25	0	0	62.5	62.5	62.5	62.5
**33**	250	50	0	12.5	12.5	12.5	12.5	125	0	31.25	31.25	31.25	31.25
**34**	250	80	0	5	5	5	5	200	0	12.5	12.5	12.5	12.5
**35**	250	90	0	2.5	2.5	2.5	2.5	225	0	6.25	6.25	6.25	6.25
**36**	250	96	0	1	1	1	1	240	0	2.5	2.5	2.5	2.5
**37**	125	0	0	25	25	25	25	0	0	31.25	31.25	31.25	31.25
**38**	125	50	0	12.5	12.5	12.5	12.5	62.5	0	15.625	15.625	15.625	15.625
**39**	125	80	0	5	5	5	5	100	0	6.25	6.25	6.25	6.25
**40**	125	90	0	2.5	2.5	2.5	2.5	112.5	0	3.125	3.125	3.125	3.125
**41**	125	96	0	1	1	1	1	120	0	1.25	1.25	1.25	1.25
**42**	25	0	0	25	25	25	25	0	0	6.25	6.25	6.25	6.25
**43**	25	50	0	12.5	12.5	12.5	12.5	12.5	0	3.125	3.125	3.125	3.125
**44**	25	80	0	5	5	5	5	20	0	1.25	1.25	1.25	1.25
**45**	25	90	0	2.5	2.5	2.5	2.5	22.5	0	0.625	0.625	0.625	0.625
**46**	25	96	0	1	1	1	1	24	0	0.25	0.25	0.25	0.25
**47**	12.5	0	0	25	25	25	25	0	0	3.125	3.125	3.125	3.125
**48**	12.5	50	0	12.5	12.5	12.5	12.5	6.25	0	1.5625	1.5625	1.5625	1.5625
**49**	12.5	80	0	5	5	5	5	10	0	0.625	0.625	0.625	0.625
**50**	12.5	90	0	2.5	2.5	2.5	2.5	11.25	0	0.3125	0.3125	0.3125	0.3125
**51**	12.5	96	0	1	1	1	1	12	0	0.125	0.125	0.125	0.125
**52**	2.5	0	0	25	25	25	25	0	0	0.625	0.625	0.625	0.625
**53**	2.5	50	0	12.5	12.5	12.5	12.5	1.25	0	0.3125	0.3125	0.3125	0.3125
**54**	2.5	80	0	5	5	5	5	2	0	0.125	0.125	0.125	0.125
**55**	2.5	90	0	2.5	2.5	2.5	2.5	2.25	0	0.0625	0.0625	0.0625	0.0625
**56**	2.5	96	0	1	1	1	1	2.4	0	0.025	0.025	0.025	0.025
**57**	1.25	0	0	25	25	25	25	0	0	0.3125	0.3125	0.3125	0.3125
**58**	1.25	50	0	12.5	12.5	12.5	12.5	0.625	0	0.1563	0.1563	0.1563	0.1563
**59**	1.25	80	0	5	5	5	5	1	0	0.0625	0.0625	0.0625	0.0625
**60**	1.25	90	0	2.5	2.5	2.5	2.5	1.125	0	0.0313	0.0313	0.0313	0.0313
**61**	1.25	96	0	1	1	1	1	1.2	0	0.0125	0.0125	0.0125	0.0125

Table S2 Fragment ion validation of trimethylsilylated and tert.-butyldimethylsilylated derivatives of aspartic acid. Aspartic acid was subjected to either trimethylsilylation or tert.-butyldimethylsilylation followed by analysis with GC-EI-MS or GC-APCI-MS. GC-EI-MS detected aspartic acid derivatives 3TMS, 2TMS and 3TBDMS. In addition, aspartic acid 2TBDMS was detected by GC-APCI-MS. Included carbon atom positions (carbon atoms) and number of labelled ^{13}C atoms (carbon atom count) were defined through analysis of positional labeled standards, [1-^{13}C], [2-^{13}C], [3-^{13}C], and [4-^{13}C]-aspartic acids, and natural compared to [U-^{13}C]-aspartic acid. Molecular formula and exact masses were predicted through in silico fragmentation analyses. Predicted exact masses were compared to measured exact masses from GC-APCI-MS analyses (mean ± standard deviation (SD) of n=3 independent experiments). Note that the mass accuracy of this study, i.e., the mean deviation of 1.3 mDa ± 0.8 mDa, was higher than previously published (Strehmel et al., 2014). The mean deviation of E^{13}C after GC-EI-MS or GC-APCI-MS analyses was determined using mixtures of positional labeled aspartic acid standards (Table S1). Thirty-six different mixtures of 25 ng total aspartic acid per injection were analyzed by at least 3 technical replicates (ND = not detected). Different ratios and isotopic concentrations were adjusted by natural aspartic acid.

Analyte	m/z	Carbon atoms	Carbon atom count	Molecular formula (predicted)	Exact mass, predicted (Da)	Exact mass, measured (Da)	Mass accuracy (mDa)	Mean deviation of E^{13}C by GC-EI-MS ± SD (%)	Mean deviation of E^{13}C by GC-APCI-MS ± SD (%)
3TMS	350	1,2,3,4	4	$C_{13}H_{32}N_1O_4Si_3$	350.1634	350.1630	0.4	ND	1.1 ± 0.6
3TMS	349	1,2,3,4	4	$C_{13}H_{31}N_1O_4Si_3$	349.1555	349.1550	0.5	-1.1 ± 2.4	ND
3TMS	334	1,2,3,4	4	$C_{12}H_{28}N_1O_4Si_3$	334.1321	334.1322	-0.1	-0.5 ± 1.8	0.2 ± 0.7
3TMS	306	2,3,4	3	$C_{11}H_{28}N_1O_3Si_3$	306.1372	306.1374	-0.3	-0.1 ± 1.4	0.0 ± 1.0
3TMS	278	1,2,3,4	4	$C_{10}H_{24}N_1O_4Si_2$	278.1238	278.1235	0.3	ND	0.6 ± 0.5
3TMS	232	2,3,4	3	$C_9H_{22}N_1O_2Si_2$	232.1184	232.1182	0.1	0.1 ± 0.6	0.7 ± 0.8
3TMS	218	1,2	2	$C_8H_{20}N_1O_2Si_2$	218.1027	218.1027	0.0	0.8 ± 2.7	0.4 ± 1.1
3TMS	205	3,4	2	$C_8H_{21}O_2Si_2$	205.1075	205.1073	0.2	4.8 ± 21.8	1.0 ± 1.0
3TMS	202	1,2	2	$C_7H_{16}N_1O_2Si_2$	202.0714	202.0716	-0.2	-9.3 ± 4.2	0.4 ± 0.9
3TMS	188	2,3	2	$C_8H_{22}N_1Si_2$	188.1285	188.1286	-0.1	-4.7 ± 2.5	0.4 ± 0.6
3TMS	160	2,3,4	3	$C_6H_{14}N_1O_2Si_1$	160.0788	160.0787	0.1	ND	0.9 ± 0.6
2TMS	278	1,2,3,4	4	$C_{10}H_{24}N_1O_4Si_2$	278.1238	278.1236	0.2	ND	-1.1 ± 1.6
2TMS	277	1,2,3,4	4	$C_{10}H_{23}N_1O_4Si_2$	277.1160	277.1158	0.2	ND	ND

2TMS	262	1,2,3,4	4	$C_9H_{20}N_1O_4Si_2$	262.0925	262.0925	0.0	0.6 ± 6.2	0.9 ± 0.7
2TMS	245	1,2,3,4	4	$C_9H_{17}O_4Si_2$	245.0660	245.0656	0.4	-10.6 ± 5.0	0.1 ± 0.7
2TMS	202	1,2	2	$C_7H_{16}N_1O_2Si_2$	202.0714	202.0719	-0.4	0.6 ± 6.2	0.0 ± 9.6
2TMS	188	1,2,3,4	4	$C_7H_{14}N_1O_3Si_1$	188.0737	188.0736	0.1	ND	2.8 ± 3.0
2TMS	160	2,3,4	3	$C_6H_{14}N_1O_2Si_1$	160.0788	160.0787	0.1	0.6 ± 1.2	0.8 ± 0.6
2TMS	130	1,2	2	$C_4H_8N_1O_2Si_1$	130.0319	130.0316	0.3	-2.1 ± 2.3	0.1 ± 1.1
3TBDMS	476	1,2,3,4	4	$C_{22}H_{50}N_1O_4Si_3$	476.3042	476.3034	0.8	ND	-5.0 ± 2.3
3TBDMS	460	1,2,3,4	4	$C_{21}H_{46}N_1O_4Si_3$	460.2729	460.2721	0.8	-0.5 ± 3.1	0.8 ± 0.6
3TBDMS	432	2,3,4	3	$C_{20}H_{46}N_1O_3Si_3$	432.2780	432.2775	0.5	-7.8 ± 6.1	0.5 ± 1.0
3TBDMS	418	1,2,3,4	4	$C_{18}H_{40}N_1O_4Si_3$	418.2260	418.2254	0.6	0.4 ± 2.4	-5.8 ± 2.7
3TBDMS	390	2,3,4	3	$C_{17}H_{40}N_1O_3Si_3$	390.2311	390.2303	0.7	-4.2 ± 2.3	1.0 ± 0.9
3TBDMS	376	1,2	2	$C_{16}H_{38}N_1O_3Si_3$	376.2154	376.2151	0.3	-6.3 ± 3.7	0.7 ± 1.3
3TBDMS	316	2,3,4	3	$C_{15}H_{34}N_1O_2Si_2$	316.2123	316.2117	0.6	-0.7 ± 1.1	0.3 ± 0.5
3TBDMS	302	1,2	2	$C_{14}H_{32}N_1O_2Si_2$	302.1966	302.1961	0.5	-0.5 ± 1.3	-0.4 ± 1.0
3TBDMS	287	1,2,3,4	4	$C_{13}H_{29}N_1O_2Si_2$	287.1731	ND	ND	2.8 ± 7.4	ND
3TBDMS	286	1,2	2	$C_{13}H_{28}N_1O_2Si_2$	286.1653	286.1650	0.3	ND	0.6 ± 0.9
3TBDMS	258	2,3,4	3	$C_{11}H_{24}N_1O_2Si_2$	258.1340	258.1342	-0.2	-3.6 ± 3.0	-1.4 ± 4.2
3TBDMS	244	1,2	2	$C_{10}H_{22}N_1O_2Si_2$	244.1184	244.1180	0.4	-7.9 ± 3.9	0.4 ± 1.1
3TBDMS	202	1,2	2	$C_9H_{20}N_1O_2Si_1$	202.1258	202.1258	0.0	-16.6 ± 11.9	-3.8 ± 25.2
3TBDMS	188	1,2	2	$C_6H_{14}N_1O_2Si_2$	188.0558	188.0556	0.2	ND	0.5 ± 1.1
3TBDMS	178	2	1	$C_5H_{16}N_1O_2Si_2$	178.0714	178.0713	0.1	ND	0.6 ± 1.2
3TBDMS	159	3,4	2	$C_7H_{15}O_2Si_1$	159.0836	159.0835	0.1	ND	-0.1 ± 1.4
3TBDMS	117	3,4	2	$C_4H_9O_2Si_1$	117.0366	117.0365	0.2	-3.5 ± 8.0	-4.6 ± 4.1
2TBDMS	476	1,2,3,4	4	$C_{22}H_{50}N_1O_4Si_3$	476.3042	476.3042	0.0	ND	-2.4 ± 2.7
2TBDMS	460	1,2,3,4	4	$C_{21}H_{46}N_1O_4Si_3$	460.2729	460.2720	0.9	ND	0.8 ± 0.7
2TBDMS	418	1,2,3,4	4	$C_{18}H_{40}N_1O_4Si_3$	418.2260	418.2254	0.6	ND	0.7 ± 0.6
2TBDMS	408	1,2,3,4	4	$C_{16}H_{42}N_1O_3Si_4$	408.2236	408.2226	1.0	ND	0.4 ± 1.0
2TBDMS	362	1,2,3,4	4	$C_{16}H_{36}N_1O_4Si_2$	362.2177	362.2178	0.0	ND	-4.4 ± 2.6
2TBDMS	346	1,2,3,4	4	$C_{15}H_{32}N_1O_4Si_2$	346.1864	346.1861	0.4	ND	1.9 ± 1.6
2TBDMS	316	2,3,4	3	$C_{15}H_{34}N_1O_2Si_2$	316.2123	316.2146	-2.4	ND	2.4 ± 3.3
2TBDMS	304	1,2,3,4	4	$C_{12}H_{26}N_1O_4Si_2$	304.1395	304.1390	0.5	ND	0.8 ± 0.8
2TBDMS	276	2,3,4	3	$C_{11}H_{26}N_1O_3Si_2$	276.1446	276.1442	0.4	ND	0.6 ± 0.6
2TBDMS	262	1,2	2	$C_{10}H_{24}N_1O_3Si_2$	262.1289	262.1286	0.3	ND	-0.6 ± 3.2
2TBDMS	216	2	1	$C_9H_{22}N_1O_1Si_2$	216.1234	216.1236	-0.1	ND	0.7 ± 2.3
2TBDMS	202	2,3,4	3	$C_9H_{20}N_1O_2Si_1$	202.1258	202.1254	0.3	ND	0.6 ± 0.5
2TBDMS	188	1,2	2	$C_8H_{18}N_1O_2Si_1$	188.1101	188.1099	0.3	ND	-3.1 ± 2.9
2TBDMS	162	2,3,4	3	$C_5H_{12}N_1O_3Si_1$	162.0581	162.0578	0.3	ND	0.5 ± 0.8
2TBDMS	158	2,3	2	$C_8H_{20}N_1Si_1$	158.1360	158.1357	0.3	ND	0.4 ± 0.6
2TBDMS	130	1,2	2	$C_4H_8N_1O_2Si_1$	130.0319	130.0316	0.3	ND	0.3 ± 1.0
2TBDMS	100	2,3	2	$C_4H_{10}N_1Si_1$	100.0577	100.0575	0.2	ND	0.4 ± 0.6

Reference

Strehmel N, Kopka J, Scheel D, Böttcher C. 2014. Annotating unknown components from GC/EI-MS-based metabolite profiling experiments using GC/APCI(+)-QTOFMS. Metabolomics 10(2): 324-336.
